# Supplementary material for: Nanoarchitecture factors of solid electrolyte interphase formation via 3D nano-rheology microscopy and surface force-distance spectroscopy
Source: Nat Commun. 2023 Mar 10;14:1321. doi: 10.1038/s41467-023-37033-7 (PMC10006426; doi:10.1038/s41467-023-37033-7)
Supplement: Supplementary file 1 — Supplementary Information [file 41467_2023_37033_MOESM1_ESM.pdf]

Supplementary Information

Nanoarchitecture factors of solid electrolyte interphase formation via 3D nano-rheology microscopy and surface force-distance spectroscopy

Yue Chen[*1, 2, 3], Wenkai Wu[4], Sergio Gonzalez-Munoz[1], Leonardo Forcieri[1], Charlie Wells[1], Samuel P. Jarvis[1], Fangling Wu[1], Robert Young[1], Avishek Dey[3, 5], Mark Isaacs[6], Mangayarkarasi Nagarathinam[7], Robert G. Palgrave[5], Nuria Tapia-Ruiz[3, 8], and Oleg V. Kolosov[* 1, 3]

[1] Department of Physics, Lancaster University, Lancaster, LA1 4YB, United Kingdom

[2] Fujian Normal University, College of Physics and Energy, 350117, Fuzhou, China

[3] The Faraday Institution, Quad One, Harwell Science and Innovation Campus, OX11 0RA, Didcot, United Kingdom

[4] College of Engineering, Swansea University, Bay Campus, Fabian Way, Swansea SA18EN, United Kingdom

[5] EPSRC National Facility for XPS (HarwellXPS), Research Complex at Harwell (RCaH), Harwell, Didcot, Oxfordshire OX11 0FA, United Kingdom

[6] Department of Chemistry, Lancaster University, Lancaster, LA1 4YB, United Kingdom

[7] Department of Chemistry, University College London, 20 Gordon Street, London, WC1H 0AJ, United Kingdom

[8] Department of Chemistry, Molecular Sciences Research Hub, White City Campus, Imperial College London, London W12 0BZ, United Kingdom

Corresponding authors: * o.kolosov@lancaster.ac.uk, yuechen@fjnu.edu.cn

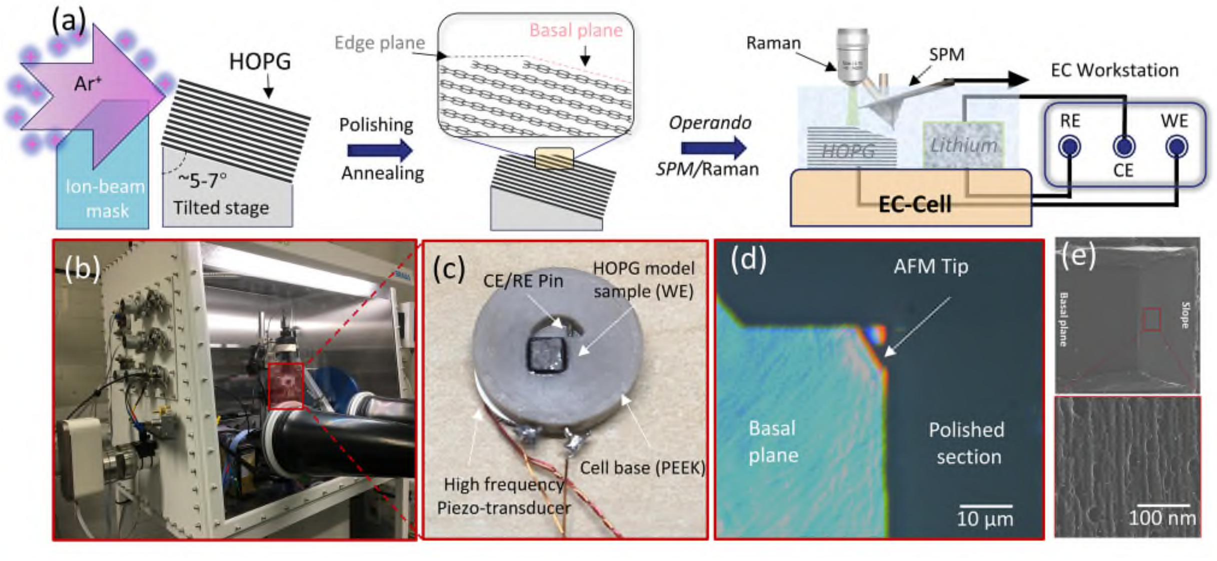

Supplementary Fig. 1 Model sample preparation and setups for EC-AFM characterizations. (a) Diagram of the sample preparation (BEXP method[1, 2]) and characterization processes. (b) *Operando* electrochemical SPM inside a glove box (see more details in the NEXGENNA project Ulab web site: https://www.nexgenna.org/?page_id=567); (c) Homemade force-modulation SPM electrochemical cell with a HOPG crystal mounted on the cell base, the high frequency piezo-transducer is used for ultrasonic force microscopy (UFM)[3, 4]; (d) Optical image of SPM tip in the boundary of sample section and basal plane; (e) SEM image on the annealed sample section (slope) and basal plane (top) and high-resolution image on section area (bottom).

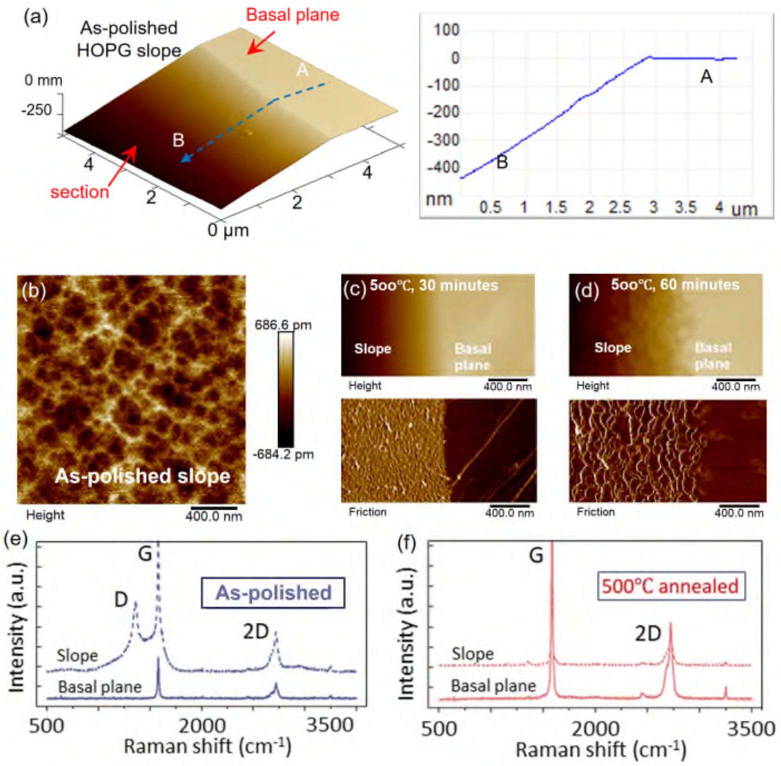

Supplementary Fig. 2 Structure of HOPG section model samples before the electrochemical cycles. (a) 3D topography image of the as-polished sample and the section cross-section line from point A → point B; (b) The "craters" on the as-polished section; (c) Topography (top) and friction (bottom) channel of the HOPG sample after 500 ℃ annealing in Air for 30 minutes; (d) The topography (top) and friction (bottom) channel of the HOPG sample after 500 ℃ annealing in Air for 60 minutes. The Raman spectra of sample (e) before and (f) after the annealing treatment. Unit (a. u.) denotes arbitrary scale.

Supplementary Note 1. The edge section topography evolution with different annealing time can be found in Figs. 2b-d. Initially, the "craters" structure can be found on the section (Fig. 2b). The annealing treatment removes these defects and changes the "nano-crater" into "fish scale" structures (Figs. 2 c and 2d), at which each scale consists of several hundreds of carbon layers. As shown in Figs. 2e and 2f, the disappeared D band in the Raman spectra of the sample section area before and after annealing treatment also confirmed that the amorphous carbon layer was removed.

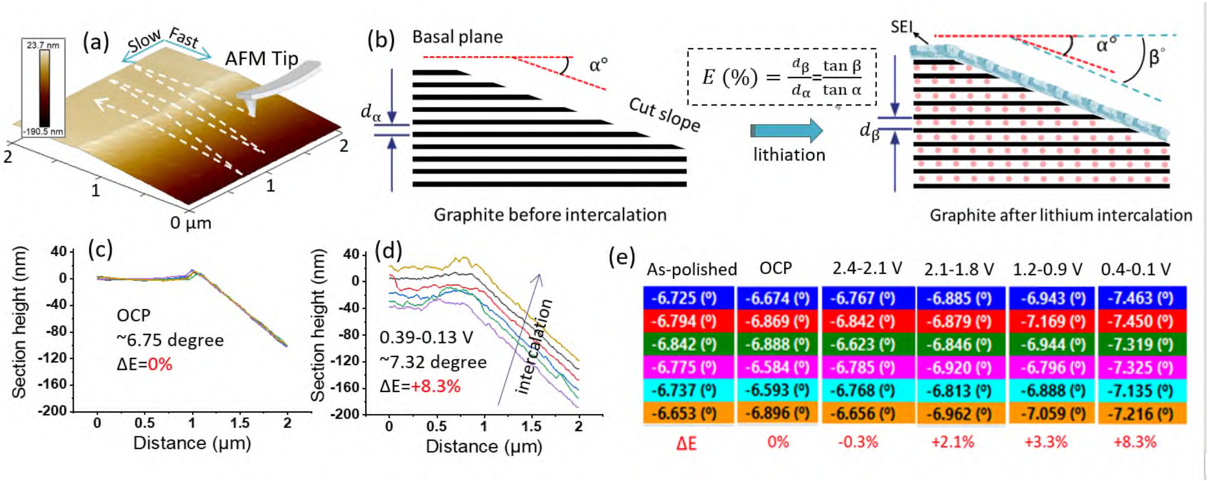

Supplementary Fig. 3 Quantification of ion-intercalation induced HOPG interlayer spacing expansions. (a) Sketch of the *operando* SPM tip scanning routes for the terrace-section profile measurements on the sample surface; (b) Calculation of the intercalation induced inter-spacing ratio (*E%*) through the terrace-section profiles, where d_α (d_β) and $\alpha°$ ($\beta°$) are the interlayer-spacing and section angle of the sample before (after) the lithium intercalation, respectively; (c) and (d) Terrace-section profiles of the HOPG sample at OCP and low intercalation voltage (0.39-0.13 V), respectively; (e) Section angle and calculated expansion ratio at different cathodic voltage during the Li-ion intercalation.

Supplementary Note 2. To correlate the SEI formation with the Li-ion intercalation, we calibrated the ion-intercalation states at different cathodic scanning voltages by measuring the lithium-intercalation induced graphite lattice expansions. As shown in Fig. 3a, we used an *operando* SPM measurement to record the sample terrace-section angle during the first lithiation process. The SPM tip's slow scanning direction is placed parallel to the terrace-section boundary, and thereby, the fast scan direction can be used to record the cross-section profiles. By measuring the sample terrace-section profile angles at different cathodic voltages, the lithium-ion intercalation can be monitored by calculating the graphite interlayer expansion rates from the section angle changes, as shown in Fig. 3b. From the geometrical relationship in Fig. 3b, one can find that the lithium intercalation induced interlayer-spacing expansion rate (Δ*E%*) can be written as:

$$\Delta E(\%) = E - 1 = \frac{d_\beta}{d_\alpha} - 1 = \frac{tan_\beta}{tan_\alpha} - 1 \quad (8)$$

Where d_α (d_β) and $\alpha°$ ($\beta°$) are the sample interlayer-spacing and section angle before (after) the lithium intercalation, respectively. *E* is the real-time inter-spacing ratio. Figs. 3c and 6d are the terrace-section profiles recorded at OCP and 0.39-0.13 V. From the figures, one can find that the lithium intercalation causes the section angle to change from the initial ~6.75 degree to ~7.32 degrees, which corresponds to an 8.3% interlayer-spacing expansion (Fig. 3e). It is worth noting that the onset of the section increase occurs at around 1.9 V which corresponds to the formation of diluted stage 1 GICs. Additionally, the ion intercalation through the section was also confirmed by *operando* Raman spectroscopy, as shown in the Raman spectra in Fig. 4. The position of the G band shifts from 1580 cm^{-1} to 1590 cm^{-1} till 0.5 V. This can be attributed to the increase of the force constants of the in-plane C–C bonds of the dilute stage 1 GICs. Moreover, the narrowing and shift of the G band are both below a potential of 0.4 V, which can be attributed to the beginning of massive Li-ion insertion. [5]

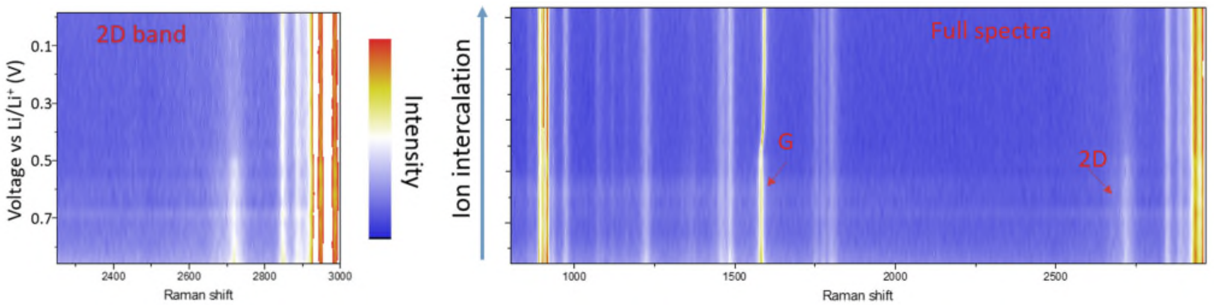

Fig. 4 Raman spectra of sample section during the 1st cathodic polarization. Graphite 2D band (left) and full range Raman spectra (right).

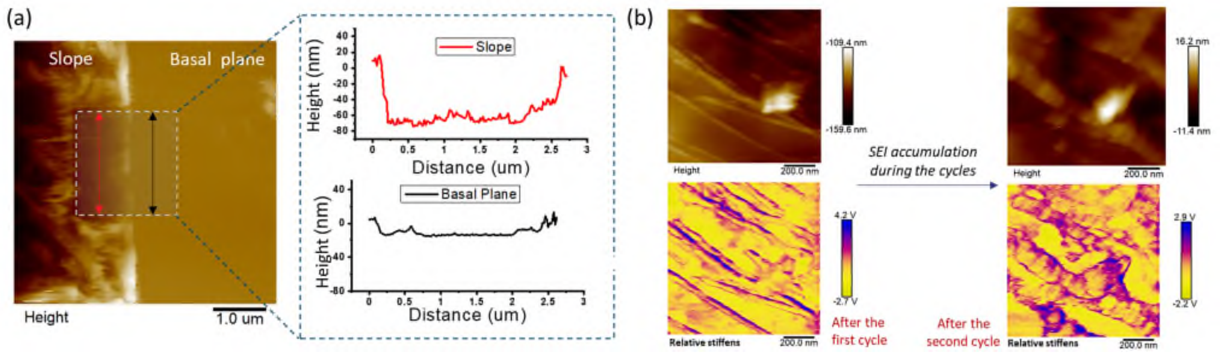

Supplementary Fig. 5 Detailed surface SEI structure characterized by EC-AFM after cycles. (a) SEI thickness measured by nano-scratching. The two cross-section profiles on the right correspond to the black (basal plane) and red (section) on the topography image on the left. (b) Nano-mechanical mapping of SEI at the edge of carbon atomic-steps (the relative stiffness is the mechanical contrast measured by ultrasonic force microscopy).

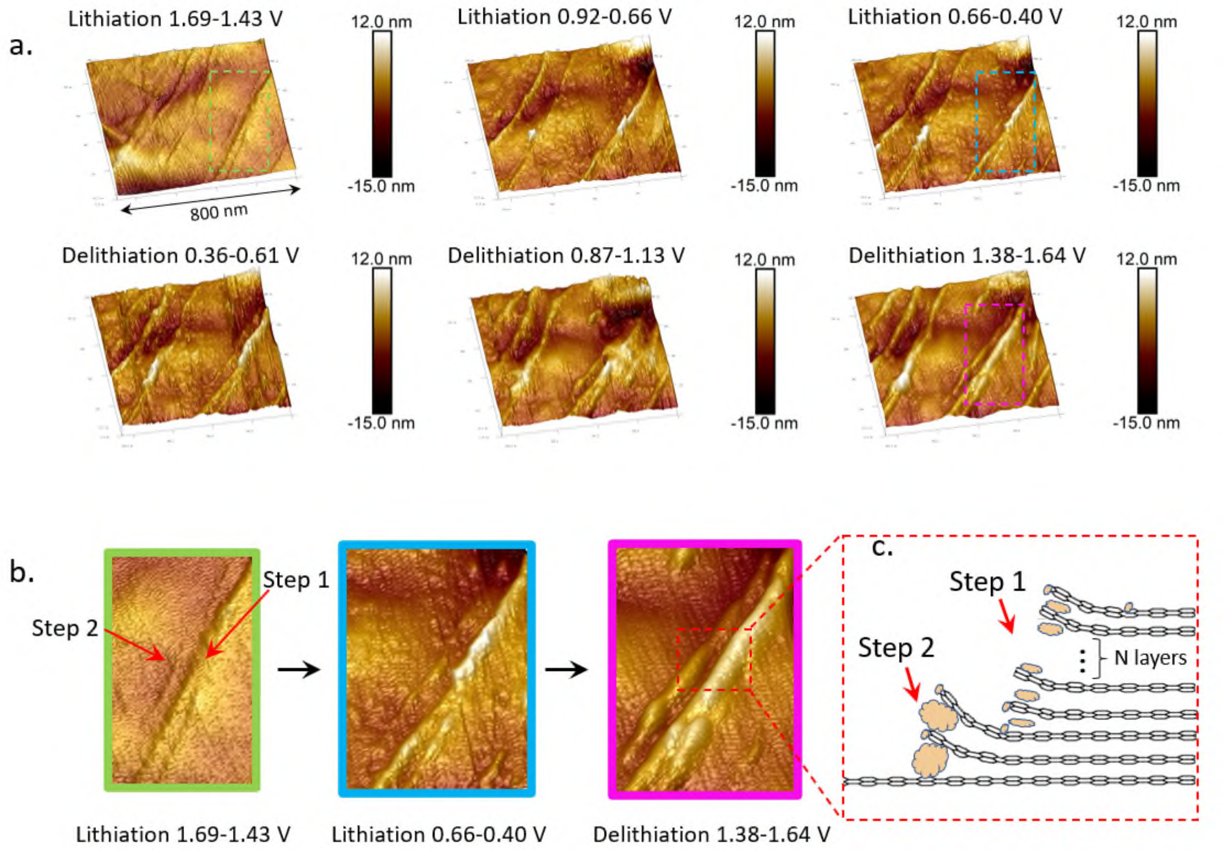

Supplementary Fig. 6 High magnification EC-AFM image showing SEI formation on basal plane and accumulation on carbon steps. (a) Three-dimensional topography images of graphite electrode surface with carbon atomic steps captured at different lithiation states. (b) High-magnification images of carbon steps during the lithiations. Three pictures correspond to the green, blue and purple dash lined square areas at different lithiation states in Fig. 6a. (c) The schematic model of SEI accumulation at a carbon atomic step[6].

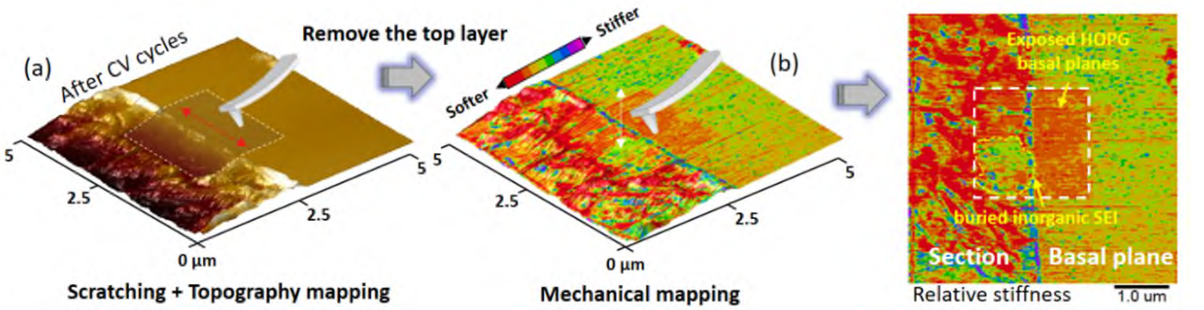

Supplementary Fig. 7 Comparation of SEI thickness and nanomechanical property on section and basal plane areas. AFM nano-scratching on the section/basal plane boundary area. (a) 3D topography and relative stiffness images. (b) 2D relative stiffness image.

Supplementary Note 3. Fig. 7 is the sample's 3D topography and relative stiffness on a 5×5 µm area after the 2nd CV cycle. By applying a higher SPM tip set force, we scratched a 2×2 µm area (containing half-section area and half terrace area) in the image centre. Then we performed the nano-mechanical mapping on the whole 5×5 μm^2 area, as shown in Fig. 7b. The area with red colour represents a softer surface, while more green/yellow colour means a stiffer surface. From the 2D mechanical image on the right (Fig. 7b), one can find that, outside of the scratched area, the section surface has lower stiffness, as well as a more inhomogeneous mechanical

distribution compared to the sample terrace surface. Additionally, inside of the scratched area, a buried inorganic SEI area (yellow dashed line area) with higher stiffness can be found at the section side; while a mechanically uniform underneath surface was observed at the sample terrace side. Thus, the nano-tomography SPM characterization confirmed that, after the CV cycles, an SEI layer with a mostly organic top-layer and inhomogeneous inorganic buried-layer, was formed on the section area. By contrast, a relatively homogenous (mosaic inorganic/organic mixed) monolayer SEI with a relatively stiffer surface was formed on the graphite basal planes.

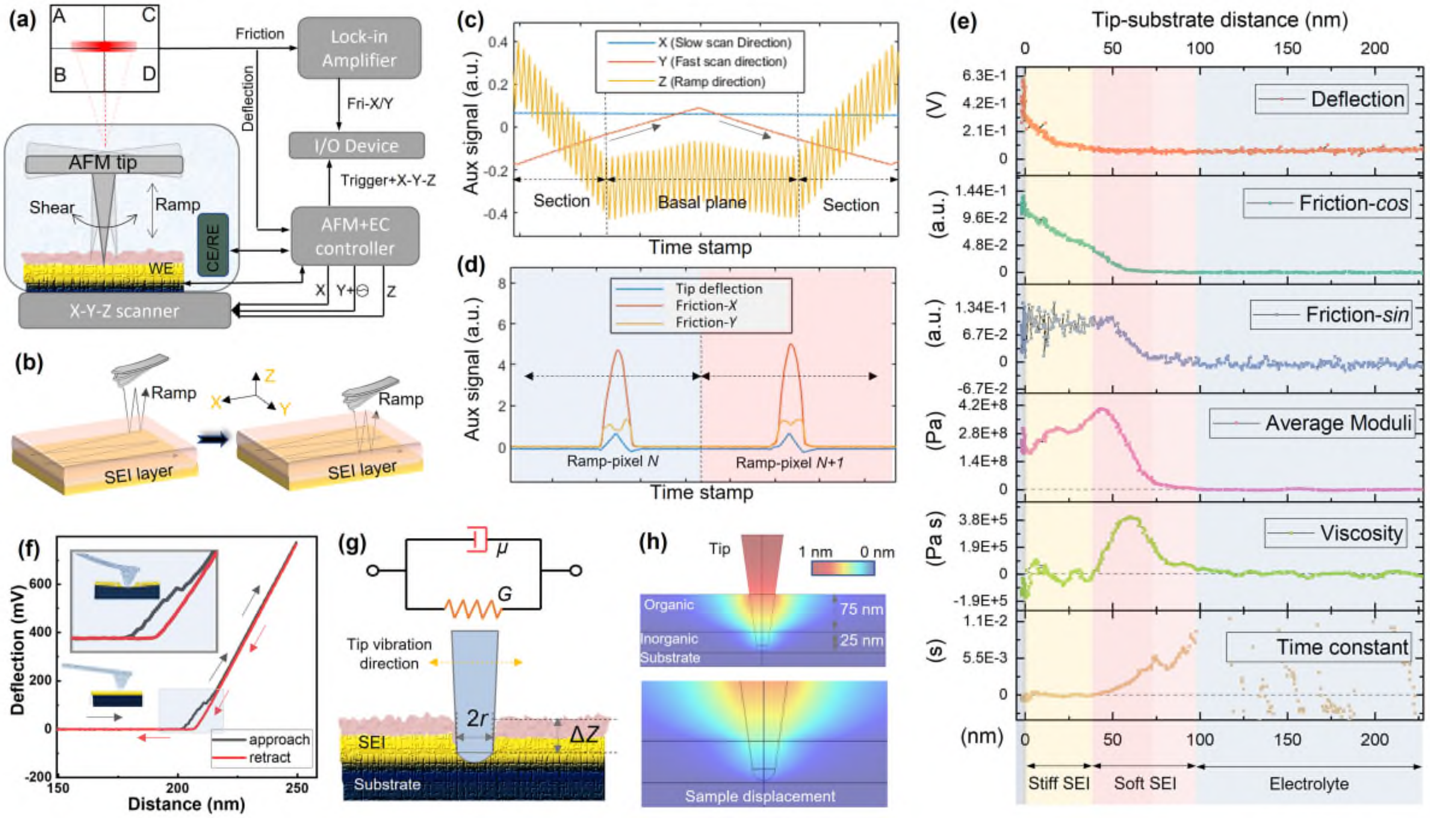

Supplementary Fig. 8 Technical details of 3D nano-rheology microscopy. Schematic diagram of (a) 3D-NRM and (b) tip movements during the measurements. (c) The real-time piezo *X-Y-Z* axis position data recorded during the 3D-NRM measurement on the polished HOPG sample during one cycle along the fast scan axis (*Y*-axis). (d) The real-time data recording of the tip-vertical deflection, shear deflection in-phase (Friction-*cos*) and out-of-phase (Friction-*sin*) signal during the two sequential ramp pixels. (e) Typical shear response spectra during the tip indentation into a multilayer SEI structure, the solid-solid contact point was set at the zero value of the tip-substrate distance (the tip indentation depth was calibrated by tip vertical deflection signal). The average elastic modulus (G), viscosity (η) and time constant are quantified by use the Friction-*cos* and Friction-*sin* force signals, respectively, according to the tip-sample contact model in Fig. 8g. (f) Typical tip deflection curve ramping on the HOPG surface in electrolyte before cyclic voltammetry cycles. (g) Tip-sample contact model. r is the radius of the tip cylinder; Z is the actual tip-substate distance (yellow and pink represent the different components in the classic dual-layer SEI structure). (h) Section view of the deformation distribution of a silicon tip dithering (2 nm peak-to-peak amplitude) in a multilayer SEI sample simulated by COMSOL. Unit (a. u.) denotes arbitrary scale.

Supplementary Note 4. To overcome the disadvantage of traditional EC-AFM that generally unable to detect the subsurface SEI structure, we developed a new microscopy technique that can provide 3D nano-scale resolution of organic and inorganic component distribution within the SEI layers under a liquid electrolyte environment. The schematic diagram of 3D nano-rheology microscopy, 3D-NRM, is shown in Fig. 8a. As shown in Figs. 8a and 8b, 3D nano-rheology microscopy is based on the combination of shear force modulation microscopy [7] and force-volume measurements. 3D-NRM senses the weak tip-sample interactions [8, 9] and viscoelastic modulus in the organic SEI components, during the force-volume ramp [10] (the tip approaches the surface from the electrolyte away from the interface, to the interface penetrating SEI layer in the *Z* direction, and mapping the *X-Y* coordinates in the raster way). Fig. 8c shows the real-time *X-Y-Z* piezo signal during the scanning back and forth along the *Y* direction (fast scan direction). As shown in the figure, the tip ramp start (*Z* position) is re-calibrated at each ramp pixel, therefore, the average *Z* position in Fig. 8c nearly follow the surfaces of the HOPG section and terrace. This is critical for keeping relative constant ramp size, avoiding the crush of tip during the ramp. During this ramp approach, we laterally dither the sample along the *X*-axis that is perpendicular to the cantilever major axis with displacement

$$\delta Y = Y_0 \cos(\omega t) \quad (1)$$

where ω is the frequency (in the range of 1-5×10^3 s^{-1}) and Y_0 (0.1-2 nanometres) is the amplitude of oscillation, respectively, then recording the in-phase (Friction-*cos*) and out-of-phase (Friction-*sin*) components of the cantilever friction signal at the drive frequency as shown in Fig. 8d. The typical spectra during one approach ramp contain the three key channels, vertical deflection, Friction-*cos* and Friction-*sin*, are depicted in Fig. 8e. The tip-sample distance in these spectra was calibrated by vertical deflection sensitivity by ramping the tip on the HOPG surface before the electrochemical cycles (Fig. 8f). The overlap area in the approach/retract cures was chosen as the deflection sensitivity calibration region regardless a native passivation layer can sometime be detected near the contact point in the approach curve. The effective modulus of SEI was quantified according to the tip-sample contact model as shown in Fig. 8g. During the measurement, the tip with a cylinder of radius r, penetrating the sample by a distance ΔZ. According to the *Kelvin–Voigt* model, the complex shear stress σ can be described as:

$$\sigma^* = G\gamma + \eta\dot{\gamma} \quad (2)$$

Where G and η are the effective shear modulus and the viscosity of the SEI layer, respectively, τ is the viscoelastic relaxation time constant, γ is the shear strain that can be approximated as

$$\gamma = \frac{\delta Y}{2r}\exp(i\omega t) \quad (3)$$

where 2r is the width of the tip cylinder along the dithering direction, and τ is the relaxation time of tip-sample junction. We can also approximate S as the effective tip-SEI contact area in the direction of the motion as:

$$S = \kappa 2r\Delta Z \quad (4)$$

Where ΔZ is the indentation depth and κ is the tip geometry factor. We choose κ=1 because the end of tip indented inside SEI layer (0~300 nm) is close to a cylinder. Then, combining Equations (1-4), the complex shear force $F^* = F' + iF''$ experienced by the stationary tip can be written as:

$$F^* = \sigma^* S = G\delta Y_0 \Delta Z(1 + i\omega\tau)\exp(i\omega t) \quad (5)$$

Fig. 8e shows the typical shear response spectrum *vs* tip-substrate distance Z during the tip penetration into the SEI layer. Upon the approach of tip to the sample surface, the AFM tip deflection channel shows only one single indentation region (tip-surface distances Z in the 0 to 35 nm range) and the tip-substrate solid-solid contact interaction region (the region around 0 nm), indicating that only the stiff SEI layer and the incompressible part of sample substrate are sensed by the tip deflection signal. By introducing the shear modulation to the tip, we observe that the Friction-*cos* (corresponding to *F'*) signal can sense the stiffer SEI layer with similar accuracy as the conventional deflection signal, whereas, significantly, now also the weaker interaction between the soft organic SEI layer and the tip is detected by the Friction-*sin* (corresponding to *F''*) response, sensing and measuring nanomechanical properties of a previously invisible viscoelastic organic SEI layer with a nanoscale resolution. From the real and imaginary parts of the *F** spectra, one can find that this SEI has a double-layer structure with a soft organic top layer extending to the electrolyte (Z range from about 40-100 nm for this sample) and a stiff inorganic underneath layer of ~40 nm thickness. By taking the derivatives of the in-phase *F*' and out-of-phase *F*'' components with respect to the penetration ΔZ in Equation (5) it is now possible to find the effective shear modulus and the viscosity of the SEI layer at the depth Z:

$$G = \frac{1}{Y_0}\frac{\partial F'}{\partial \Delta Z} \quad \text{and} \quad \eta = G\tau = \frac{1}{Y_0 \omega}\frac{\partial F''}{\partial \Delta Z} \quad (6)$$

With a calibrated tip lateral force constant, the G values of these inorganic/organic layers and the viscosity η value for the organic layer can be quantified as shown in the Fig. 8e. The details of calibration and verification of this model can be found in the following Note 5. The significant advantage of shear modulation in 3D-NRM is that it eliminates the effects of the substrate. As shown in the finite elements simulation (Fig. 8h), as the elastic moduli of the SEI are smaller than the ones of the substrate (electrode material), the shear dithering with an peak-to-peak amplitude of 2 nm generates the lateral deformation exclusively within the SEI layer. This allows to accurately probe the nanomechanical properties of the SEI by eliminating the interference of the anode material that is inevitable in the conventional nano-indentation method[11]. By reconstructing the obtained mechanical properties in the three dimensions, 3D-NRM allows to further obtain a full nanoscale picture of the elastic and viscoelastic components distribution inside the SEI, with the thickness (Z coordinate) resolution of about 5 nm and the lateral (X-Y) resolution of 10-20 nm.

Supplementary Note 5. 3D reconstructed images rendered using values of relaxation time constant τ (or shear phase) can further reveal the internal structure of this SEI layer. According to Equation (5), tip shear amplitude (A) and phase (ϕ) are linked with the shear modulus G and the relaxation time constant, τ, as:

$$A = Y_0 \Delta Z\, G\sqrt{1+(\omega\tau)^2} \quad \text{and} \quad \phi = \tan^{-1}(\omega\tau) \qquad (7)$$

The ω and Y_0 are constant values set during the measurement, whereas the ramp frequency at each pixel point is much smaller than the drive frequency ω, allowing to safely neglect the *non-Newtonian* behaviour of the SEI. For the uniform SEI layer, one can find that the tip shear forces are increasing approximately linearly with the indentation depth ΔZ, while the ϕ is generally independent of ΔZ and therefore reflects the viscosity/elasticity ratio of the tip-SEI junction during the indentation at the excitation frequency. This enables the phase image to differentiate the inhomogeneity inside the SEI layer along the SEI depth. As shown in Fig. 9a, the viscous SEI layer (yellow), which causes the shear phase delay, on the stiff electrode surface (purple) can be well-differentiated with nanoscale resolution in the 3D reconstructed phase mappings. Besides, this 3D reconstruction mapping can be performed *in situ* (Fig. 9b), providing a snapshot of localized nano-structure relative SEI information on the electrode surface in the liquid electrolyte environment.

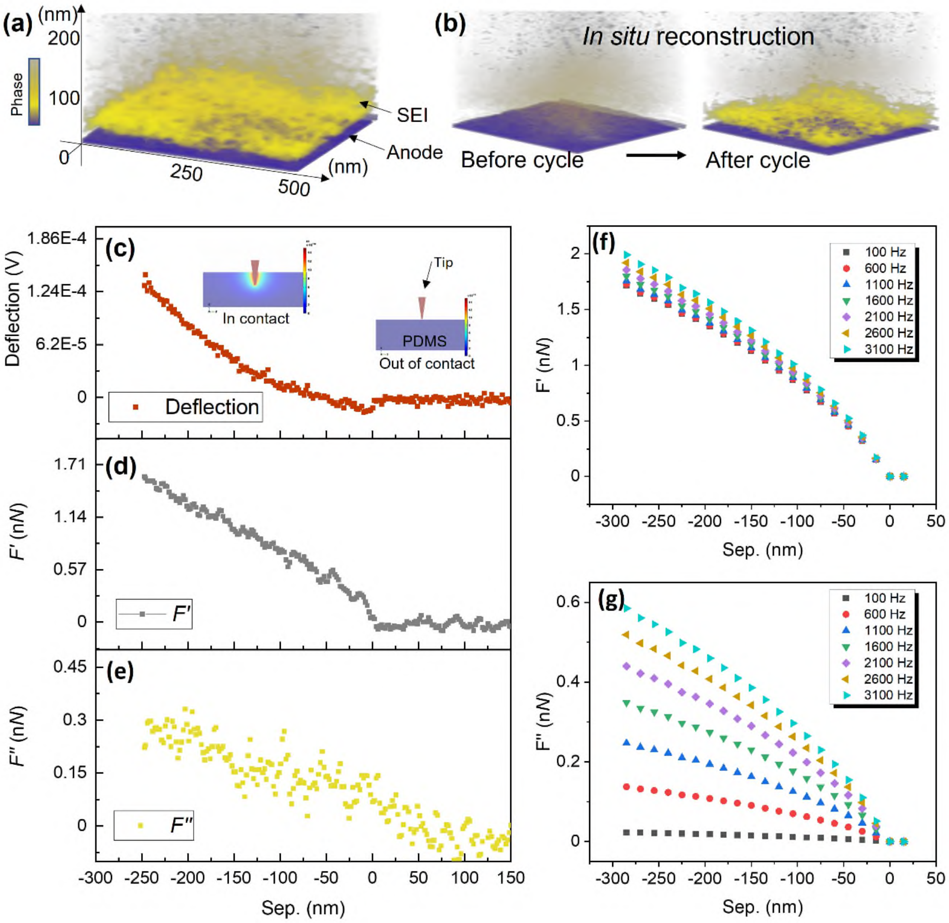

Supplementary Fig. 9 Typical reconstructed SEI structures measured by 3D-NRM and force calibration results in PDMS standard samples. (a) 3D reconstruction mapping of the shear phase of SEI formed on HOPG surface. (b) 3D shear phase mapping measured by *in situ* 3D NRM before and after the electrochemical cycles. Typical tip approach curves in PDMS (PDMS sylgard-184) measured using nano-rheology microscopy: (c) tip deflection (insets are the COMSOL simulation results of the displacement section of Tip-PDMS conjunction), (d) tip shear force in-phase component (elastic) and (e) out-of-phase component (viscous). The tip-PDMS contact point was set as the zero-separation point. COMSOL simulation of (f) elastic response and (g) viscous response of tip-PDMS junction at different indentation depths and under various shear vibration frequencies. The key simulation parameters adapted from the PDMS calibration experiments are maximum tip indentation depth 300 nm, modulation frequency 0.1-3.1 kHz, peak-to-peak amplitude 2 nm.

The PDMS calibration sample was purchased from Sigma and fabricated according to the instructions. After mixing the base and binder, the slurry was spin-coated on a glass substrate at 1500 RPM. Figs. 9c-e is the typical spectra of tip deflection, shear force *F'* and *F''* components measured in the PDMS calibration samples. The tip-PDMS contact point was set as zero separation point. The normal and lateral tip force constant was calibrated according to the Sader method and cantilever geometry[12], respectively. As shown in Figs. 9c-e, at test shear frequency (1.32 kHz), with the increasing of the tip indentation (ΔZ) during the approach, the *F'* and *F''* increase with the increase of indentation depth. According to Equation (6), the average shear modulus *G* and the viscosity η of the PDMS can be obtained by fitting the linear slope value of *F'*-(Sep.) and *F''*-(Sep.), respectively. The obtained values are about $G \approx 4.04$ MPa, $\eta \approx 818$ Pa s, which are consistent with the reported values [13 14]. By adapting the measured rheology mechanical values of PDMS, we constructed a tip-PDM contact model to simulate and verify the *F'*-(Sep.) and *F''*-(Sep.) calibration force spectra. Tip amplitude was set as 2 nm (peak-to-peak). *Kelvin-Vogt* model was chosen to generate the viscosity of PDMS in the contact mechanical simulation. The tip geometry, indentation depth and other parameters were set according to the PDMS calibration experiments. The frequency was swept from 100 Hz to 3100 Hz. Figs. 9f and 9g are the COMSOL simulation results of *F'* and *F''* spectra at different shear vibration frequencies. As shown in figures, comparing to the experimental measured force curves, the obtained simulation curves at various frequency show same dependence with increase of the sample separation (tip indentation), as well as the close value of tip force (with an error less than 10% at tip each separation position for both *F'* and *F''*), indicating that our 3D-NRM measurements are able to precisely determine the absolute shear modulus and viscosity of the samples.

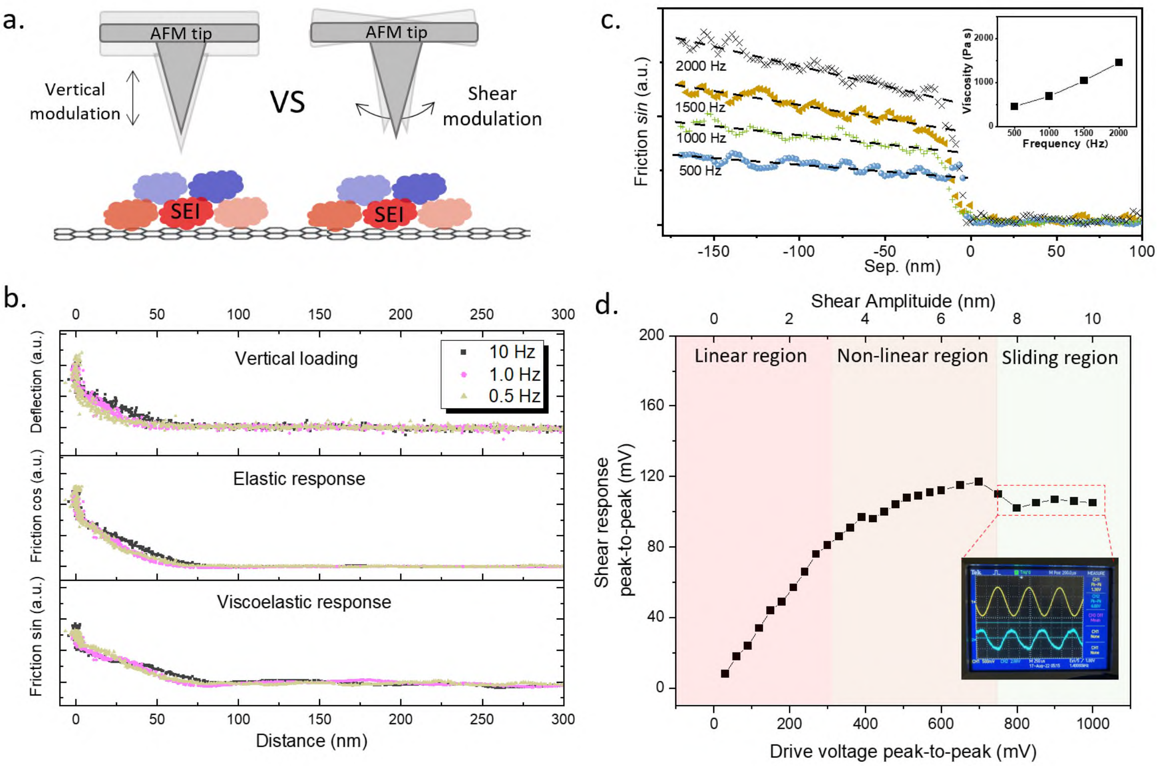

Supplementary Fig. 10 The effects of vertical loading speed/frequency and lateral (shear) modulation frequency on the sample mechanical quantification. (a) Schematic comparison of traditional vertical force modulation (left) and small amplitude shear force modulation (right) in this work. (b) Tip deflection (vertical loading), elastic (Friction *in-phase*) and viscose (Friction *out-of-phase*) response signals were recorded at different vertical loading speeds on the as-formed SEI. The shear modulation frequency is about 1250 kHz. (c) Viscosity component signal (Friction *out-of-phase*) at different shear modulation frequencies in PDMS calibration sample. (d) The dependence of cantilever response signals and shear modulation amplitudes when tip and sample are in the solid-solid contact point. In the inset, the yellow and blue sinusoidal

curves are driving and response signals displayed in an oscilloscope, respectively. Unit (a. u.) denotes arbitrary scale.

Supplementary Note 6. The effects of vertical loading speed/frequency and lateral (shear) modulation frequency on the sample mechanical quantification and resolution is evaluated. As shown in Fig. 10a, 3D nano-rheology uses small amplitude shear modulation, instead of traditional vertical force modulation, to avoid the crosstalk of vertical indentation/mechanical response to the lateral shear response of the AFM tip. For the force volume AFM model[10, 15], the typical vertical loading speed is in the range of ~0.5-10 Hz (corresponding to the tip velocity of 4000-200 nm/s for a 200 nm ramp size). Within this narrow loading frequency range, the tip indentation-induced SEI deformation pit can relax to the equilibrium state at each indentation depth, and therefore the *non-Newtonian* effects can be safely neglected. As shown in Fig. 10b, the measured lateral elasticity and viscosity signals at different loading frequencies are almost overlapped, barely depending on the tip vertical loading speed, indicating the vertical loading speed has less effect on the vertical resolution. But it should be noted that the low loading speed could also take a longer time for the 3D imaging process. Meanwhile, we also noticed that the effective elastic response measured by the shear modulation signal is largely dependent on the modulation frequency, as would be expected from the viscoelastic organic part of SEI. As shown in Fig. 10c, within the instrument accessible shear modulation frequency range (~500-2000 Hz), the out-of-phase signal increase with the modulation frequency, and the measured viscosity of PDMS sample shows positive proportional to the modulation frequency (inset in Fig. 10c). Besides, it is also found that the viscosity response shows a higher signal-to-noise ratio in higher modulation frequency under optimized data aquation time constant. To optimise the experiment conditions, the modulation frequency was selected at the ~kHz range according to the material relaxation time constant and avoiding the instrument resonance frequencies. Last, the shear modulation amplitude in our experiment is limited to around 0.1-2 nm (peak-to-peak), small drive amplitude generates a weak tip torsional signal that may out of the photodetector sensitivity, while large shear amplitude modulation causes the non-linearity of response or sliding on sample surface as shown in Fig. 10d. In the non-linearity response, the concurrent cantilever torsional & bending are detrimental to the calibration of the force constant[12]. Moreover, under large shear modulation amplitude, the tip apex stirs inside SEI, reducing the in-plane resolution.

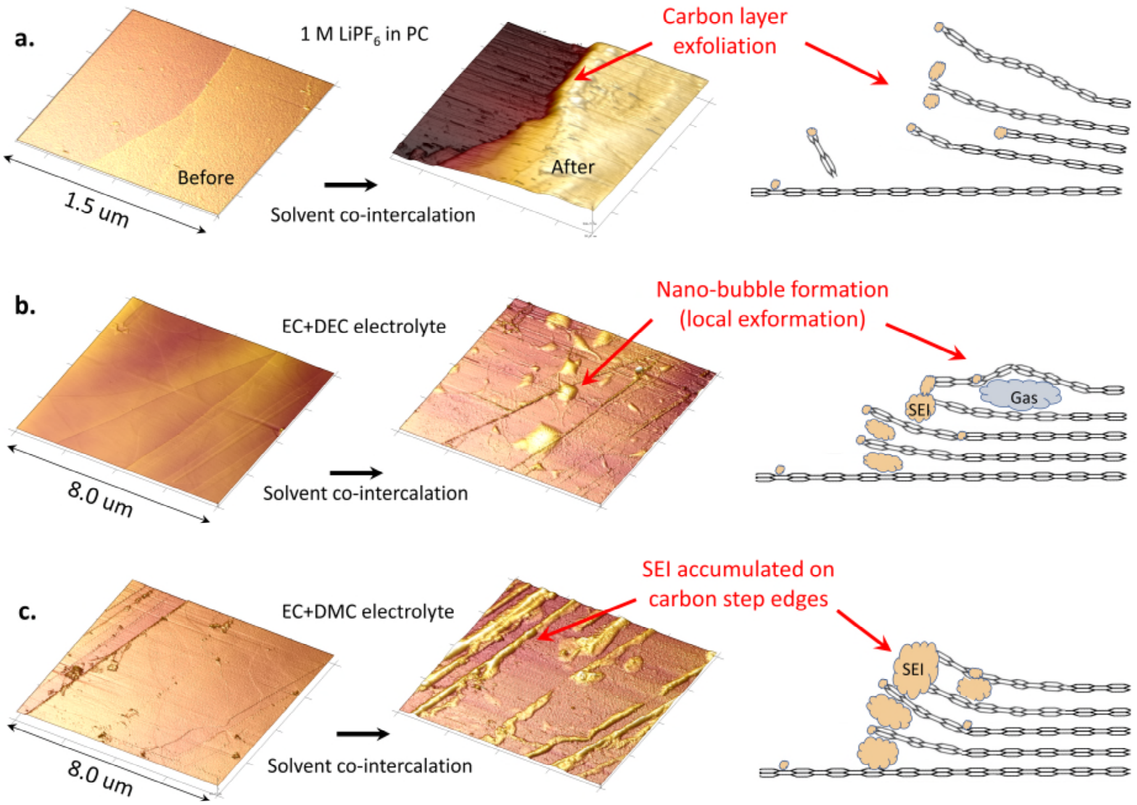

Supplementary Fig. 11 Changes of HOPG-electrolyte interfacial nanostructure after lithiation in various electrolytes. Three-dimensional surface topography of HOPG surface with carbon atomic steps before and after the solvent co-intercalation/decomposition in 1M $LiPF_6$ (a) PC, (b) EC: DEC=1:1 (v/v) and (c) EC: DMC=1:1 (v/v) electrolytes.

Supplementary Note 7. Fig. 11 summarized the different solvent co-intercalation effects on our model sample. In the early stage of lithiation, the electrolyte containing PC solvent causes the notable carbon layer expansion/exfoliation due to PC co-intercalation (Fig. 11a); the electrolyte containing DEC co-solvent can also co-intercalate and have slightly weaker the Van der Waals interaction with carbon layers, generating many nano-blisters/bubbles trapped inside graphite[16, 17] (Fig. 11b), which is detrimental for the mechanical property quantification of later formed SEI layers; In EC/DMC mixed electrolyte, the solvent co-intercalation and sequential decomposition effectively sealed the carbon step edges[6] and forms carbon edge wrapping at initial lithiation stage (Fig. 11c), preventing the further graphite exfoliations/delamination. EC/DMC passivated HOPG surface can thereby serve as an ideal “solid-substrate” for the study of nanoscale mechanical property of SEI layers. We therefore selected the weakly solvated solvent which has distinct solvation structures with lithium ions[18-20], to eliminate the solvent co-intercalation triggered carbon layer exfoliation and sample damage[21].

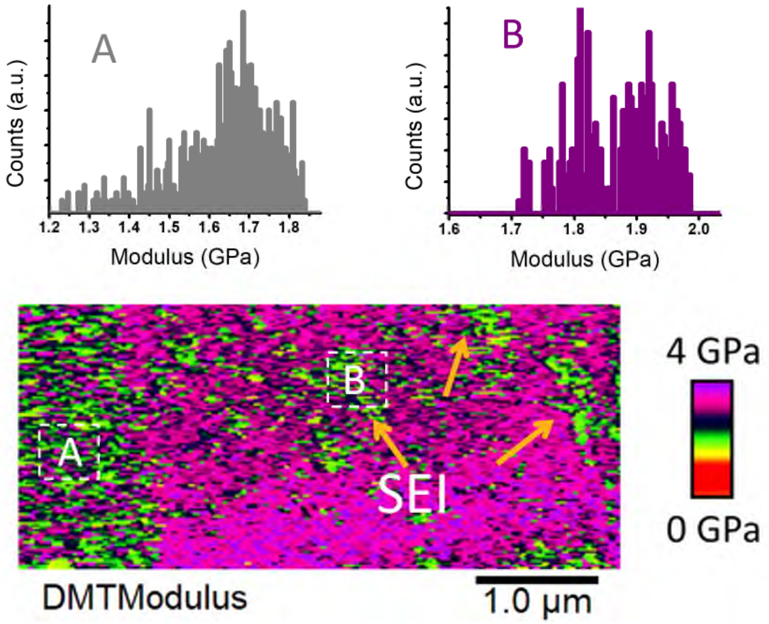

Supplementary Fig. 12 Nanomechanical properties of SEI formed in DX-based electrolyte. DMT modulus of the SEI on the section and basal planes during 0.7-0.6 V in DX-based electrolyte. Unit (a. u.) denotes arbitrary scale.

Supplementary Note 8. Fig. 13a shows the full survey spectra of SEI formed at section and basal plane in DX-based electrolyte, and the quantified atomic percentage of Li, O, C, F, S and N elements are depicted in the pie chart on the right panel. It is found that the SEI components formed in DX are similar at the sample section and basal plane, namely, these SEI components are independent of graphite crystal planes. Both SEIs contain around ~12.7-13.5 At% Lithium, ~23.6-25.5 At% Fluorine, ~9.7-10.2 At% Oxygen, ~47.7-52.4 At% Carbon, and a small amount of ~1.1-1.8 At% Sulphur and ~0.5-1.4 At% Nitrogen. The detailed high-resolution spectra of each element on the sample basal plane and section can be found in Figs. 13b-g and S7.1b'-g'. The deconvolution of these high-resolution spectra confirmed the identical SEI components on two graphite crystal planes, including LiF, Li_3N, sulphide and carbonate organic species. In the *C 1s* spectra in Figs. 13b and 13b', excepting the strong C-C peak (partially contributed by the exposed graphite electrode), the organic species-related peaks (C-O/C=O/C-F) are much smaller than the peaks observed in the EC based electrolyte, indicating much less carbonate organic species are formed on the SEI in DX-based electrolyte. This is consistent with the negligible organic fluoride peak as shown in *F 1s* spectra in Figs. 13c and 13c'. Interestingly, *O 1s* spectra contain a large $S\text{-}O_x$ species-related peak at a binding energy of ~531.5 eV, which can be attributed to the six-electrons decomposition product ($Li_2S_2O_4$) of FSI^- anions[19]. The existence of $Li_2S_2O_4$ and other sulphide is also confirmed by the $S\text{-}O_x$ specious related peaks at ~167 eV in the *S* $2p_{3/2}$ spectra (Figs. 13e and 13e'). Further examining the *Li 1s* and *N 1s* peaks in Figs. 13f-g', a small amount of Li_3N was also suggested in the SEI. Therefore, from the *O 1s*, *S 2p* and *Li 1s* spectra in Fig. 13, one can conclude that the SEI component formed in DX based electrolyte contains many inorganic species such as LiF, $Li_2S_2O_4$ and Li_3N[18, 19] which derive from the decomposition of FSI^-.

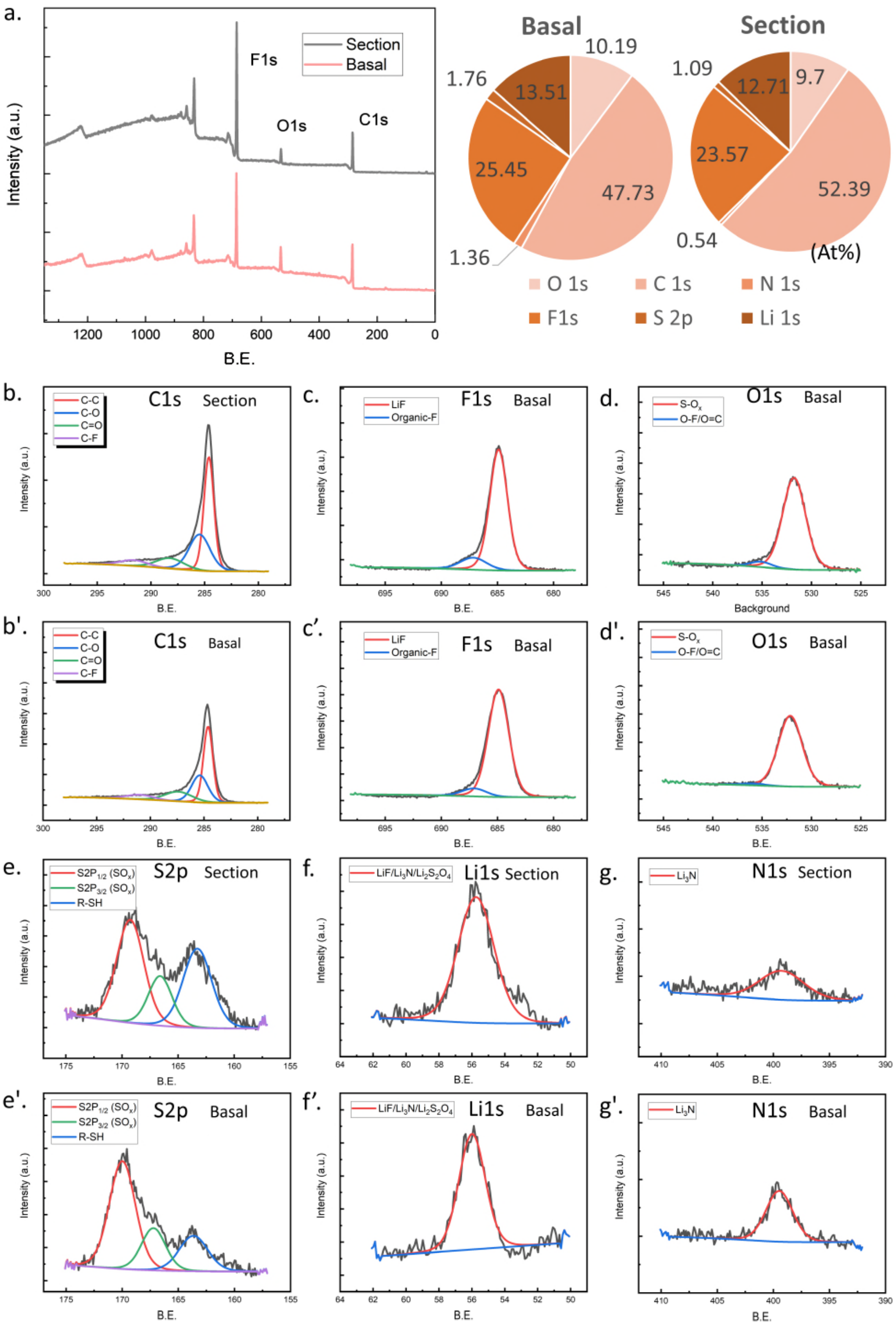

Supplementary Fig. 13 XPS measurements of SEI formed in DX-based electrolyte at sample section area and basal planes. (a) XPS survey spectra and quantifications of the elemental atomic percentage. (b, b') *C 1s*, (c, c') *F 1s*, (d, d') *O 1s*, (e, e') *S 2p*, (f, f') *Li 1s* and (g, g') *N 1s* high-resolution spectra of SEI layer formed in DX electrolyte. Unit (a. u.)

denotes arbitrary scale.

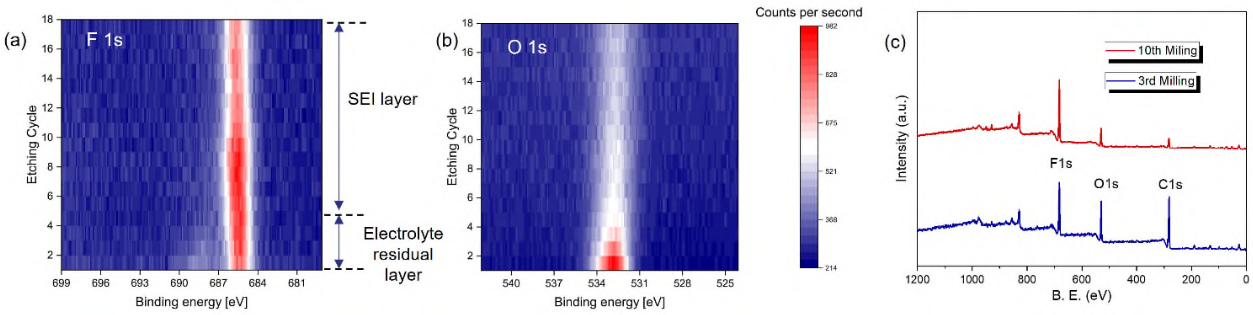

Supplementary Fig. 14 XPS depth profiling spectra of the as-disassembled HOPG anode surface in DX-based electrolyte. XPS depth profiling in the (a) *F 1s* and (b) *O 1s* regions. (c) Example survey spectra collected after the 3rd and 10th milling cycle. Unit (a. u.) denotes arbitrary scale.

Supplementary Note 9. Figs. 14a and 14b are the *F 1s* and *O 1s* spectra during the XPS milling. From the figure, we observe the electrolyte residual layer which is removed following about six milling cycles. Following this, the *O 1s* peak intensity decreases significantly, while the *F 1s* stays relatively constant, indicating that the SEI formed in DX-based electrolyte contains more fluoride, rather than oxide. This implies that the LiF, rather than the Li_2O or $LiCO_2$, is the main inorganic components in the SEI layer formed in DX-based electrolyte. Moreover, a much higher *F 1s* peak intensity compared to the *C 1s* can be found in the survey spectrum in Fig. 14c within the SEI layer, further indicating a high proportion of inorganic fluoride in the SEI.

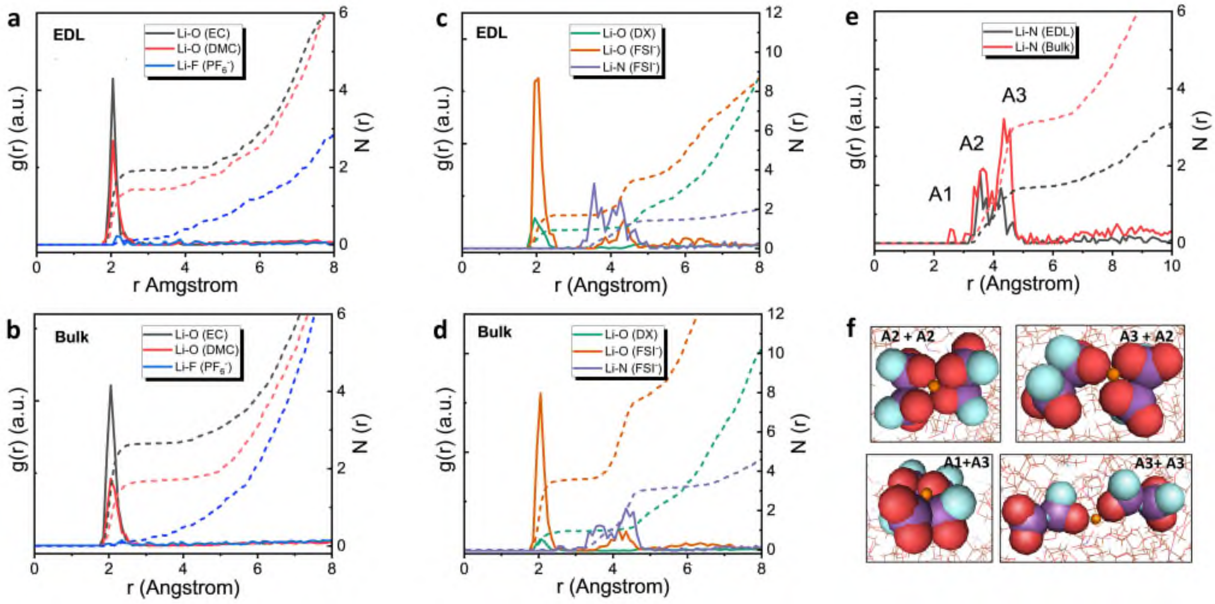

Supplementary Fig. 15 Lithium-ion's solvation structures in EC- and DX-based electrolytes under the negatively charged electrode condition. Radial distribution function (RDF) and coordination numbers (CN) of Lithium in (a, b) EC and (c, d) DX based electrolyte inside and outside of EDL. (g(r) functions inside and outside (bulk) the EDL were calculated by $0<Z_{EDL}<2$ nm, and $Z_{Bulk} > 2$ nm according to the experiment measured force-distance curves) (e, f) Three types of Li-FSI solvation structures inside EDL of DX based electrolyte. Small brown ball is Li^-, large-side spherical structure ions are FSI^-, stick structure in the background are other solvent molecules/ions. Unit (a. u.) denotes arbitrary scale.

Supplementary Note 10. Apart from the density distribution profiles along the Z direction, we further consider EDL structure at finer level focusing on the difference of lithium solvation structure of bulk electrolyte and near electrode surfaces under the negatively charged surface. Figs. 15a and 15b show EDL and bulk electrolyte RDFs for the carbonyl oxygen of EC and DMC, the F atoms of PF_6^- anion with lithium-ions. In EC-based electrolyte, examination of the oxygen atom distributions near the surface and bulk shows that the magnitude of the first peak in density of EC oxygen atoms reduced inside EDL due the partial adsorption of lithium on electrode surface. The average coordination number (CN) of EC is slightly decreased, but the first peak for DMC oxygen stays nearly constant. This is due to the flexible chain structure of DMC that can self-rearrange inside EDL. A significant drop of F coordination number is observed inside the EDL, indicating that PF_6^- anions are repelled from the innermost EDL. This EC/DMC solvent dominating solvation structure is consistent with previous reports[22, 23] and further confirms the solvent preferential decompositions. At the same time, in the DX-based electrolyte (Figs. 15c and 15d), although a decrease of FSI^- coordination number (from about 3 to 2) is observed inside the EDL under the negative polarization, the FSI^- anion still dominates the first solvation shell with higher CN number than DX solvent, indicating the anion dominated solvation structure is preserved. Interestingly, the FSI^- disorbtion or reorientation away from electrode surface upon charging was also observed. As shown in Figs. 15e and 15f, the FSI^- coordination structures can be classified in three types[24]: A1 (Li^+ coordinates to the N atom), A2 (bidentate, Li^+ coordinates to oxygen atoms on both sides of the N atom) and A3 (monodentate, Li^+ coordinates to oxygen atoms on one side of the N atom), corresponding to the three RDF peaks between 2-5 Angstrom in Fig. 15e. Significant decrease of A1 and A3 peaks in the Li-N RDF are found inside the EDL, indicating the Li^+ coordinates to oxygen atoms in A2 mode is the preferential coordination structure on a negatively polarized electrode surface. This narrowed distribution function of Li-FSI^- coordination facilitates the ordered molecule/ion packing structure on the electrode surface, which is consistent with the oscillated atomic force curves observed in Fig. 4b of main manuscript. Moreover, during the initial polarization, the rearrangement and de-coordinated FSI^- from A1/A3 structure, which has lower binding energies than the A2 structure, can be the indication of the initial SEI formation, facilitating the preferential decomposition of anion and forming a fluorine rich inorganic SEI layer.

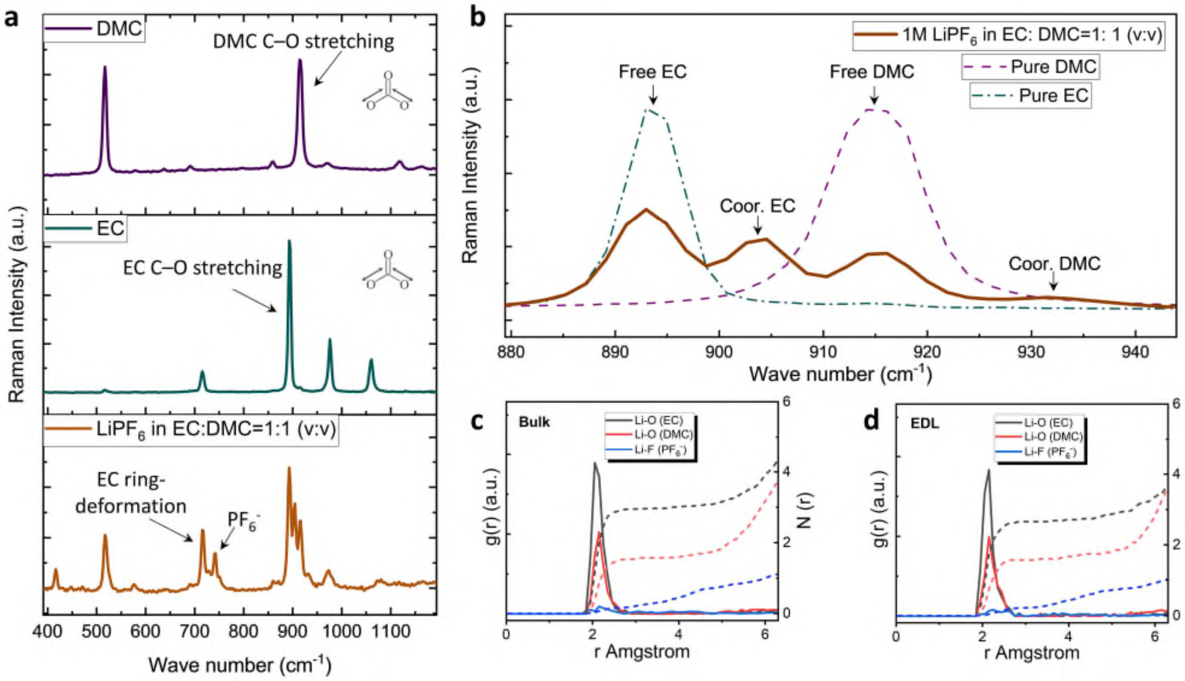

Fig. 16 Lithium-ion's solvation structures in EC-based electrolytes without negatively charging the electrode. (a) and (b) Raman spectra of pure EC, DMC and 1M LiPF6 in EC: DMC=1:1 electrolyte. (c) Radial distribution function of Li-O (EC), Li-O (DMC) and Li-F from MD simulation results in 1M $LiPF_6$ in EC: DMC=1:1 electrolyte without negatively charging the electrode. (g(r) functions inside and outside (bulk) the EDL were calculated by $0<Z_{EDL}<2$ nm, and $Z_{Bulk} > 2$ nm according to the experiment measured force-distance curves). Unit (a. u.) denotes arbitrary scale.

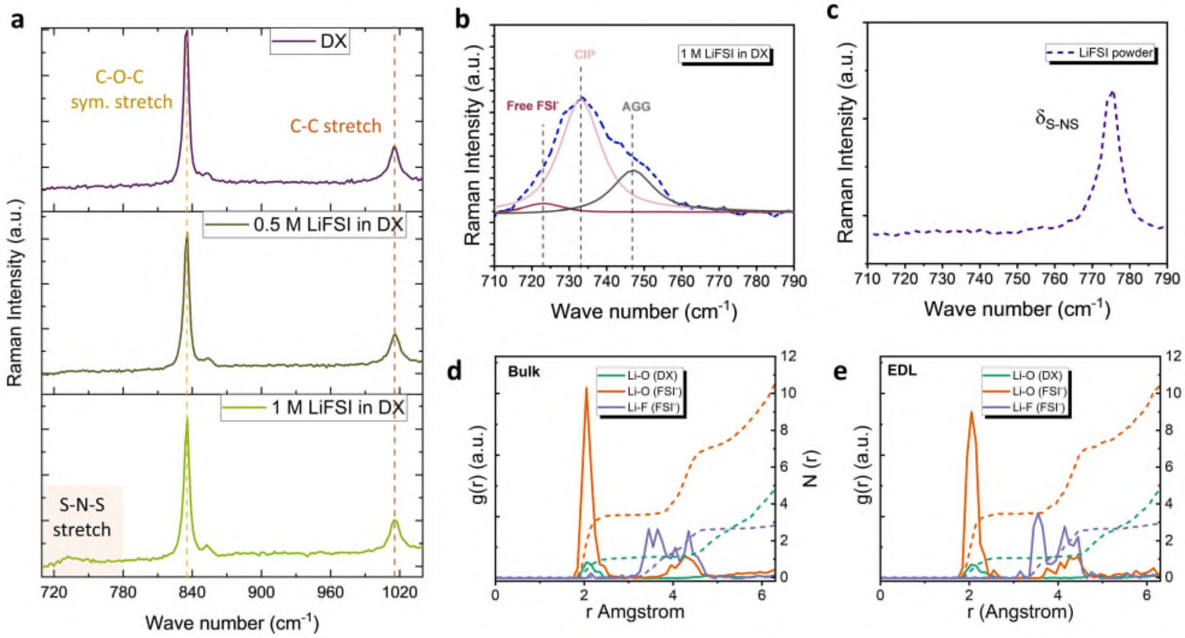

Supplementary Fig. 17 Lithium-ion's solvation structures in DX-based electrolytes without negatively charging the electrode (a) Raman spectra of pure DX, 0.5 and 1M LiFSI in DX electrolyte. (b) Deconvoluted S-N-S stretch band of 1M LiFSI in DX electrolyte and (c) the S-N-S stretch band in LiFSI powder. (d) RDF of Li-O (DX) and Li-O (FSI^-) from MD simulation results in 1M LiFSI in DX electrolyte without negatively charging the electrode. (g(r) functions inside and outside (bulk) the EDL were calculated by $0<Z_{EDL}<2$ nm, and $Z_{Bulk} > 2$ nm according to the experiment measured force-distance curves). Unit (a. u.) denotes arbitrary scale.

Supplementary Note 11. We performed Raman spectroscopy measurements and MD simulations to understand the solvation structures in our bulk electrolytes. As shown in Figs. 16a-c, pure EC and DMC solvents show a strong C-O stretching vibrational band at ~893 cm^{-1} and 903 cm^{-1}, respectively, while in 1 M $LiPF_6$ EC: DMC=1:1 electrolyte, these characteristic vibrational peaks split into another satellite peak at higher wave number at ~915 cm^{-1} for EC and ~933 cm^{-1} for DMC, corresponding to the solvent coordination interaction with lithium ion[25-28]. The I_{Coor}/I_{Free} of DMC (~0.21) is much smaller than EC (~0.71), indicating preferential coordination of EC solvent to lithium ions compared to DMC. It is worth noting that, although some reports suggested the cyclic carbonate solvents are the preferred members of the Li^+-primary solvation sheath and neither salt species (PF_6^+ in this work) nor their concentrations have a significant impact on this trend[29], the coordination of DMC is still non-negligible in our electrolyte giving the existence of the broad DMC coordination band at ~933 cm^{-1}. Our MD calculated RDF (Figs. 16d-e) also shows that each lithium is surrounded predominantly by EC, while solvated by at least 1~2 DMC and one PF_6^+ inside the first solvation shell. This is consistent with previous Raman[25, 30], IR[31, 32], SIMS[33], and NMR[34] studies which support such arrangement. While the primary solvation sheath of Li^+ is generally occupied by cyclic carbonate molecules (EC), the linear carbonate (DMC) with relatively weak, but persistent interaction with lithium ions, could still stay in the first solvation sheaths[35]. This coordination of solvents with lithium can alter the electrolyte LUMO energy level and effectively modulate the solvent reductive decomposition stability[36, 37].

Interestingly, such a domination of solvents in the first solvation sheaths can be broken when low permittivity/non-solvating solvents, such as benzene and 1,4 Dioxane (DX), are used in the solution system[18, 38]. As shown in the Raman spectra in Fig. 17a, the fingerprint peaks of DX solvent do not shift in 0.5 M and 1 M LiFSI electrolytes, especially the peak position and symmetricity of the C-O-C symmetric stretch band (~ 842 cm^{-1}) [39] kept constant (no satellite peak appears) after adding LiFSI, indicating a weak carbonyl oxygen coordination with lithium ions. With the increase of salt concentration, the S-N-S stretch-related peak (~ 720-760 cm^{-1}) from FSI^- anions starts to appear in the Raman spectrum of 1 M LiFSI electrolyte. This S–N–S stretch band was deconvoluted into three distinctive bands (Fig. 17b): free anion (~721 cm^{-1}, non-coordinated FSI^-), contact ion pair (CIP, 733 cm^{-1}, one FSI^- binding with one Li^+) and ion aggregates (AGG,745 cm^{-1}, one FSI^- binding with two or more Li^+)[18, 40, 41]. In DX-based electrolyte with a low dielectric constant solvent, the ratio of free anion is much smaller than the ratio of CIP and AGG. We also noticed that the S–N–S Raman shift of AGG is only ~31 cm^{-1} away from the S–N–S vibration mode in the LiFSI powder at ~ 776 cm^{-1}[42] (Fig. 17c), indicating a strong coordination interaction of FSI^- with lithium ions in DX-based electrolyte. This is consistent with our MD simulated solvation structure, in which each lithium was preferred solvated by three FSI^- anions instead of DX solvents as shown in the Li-O RDF in Fig. 17d. Therefore, in the tentative sketch the lithium-ions are mainly solvated by solvents in EC-based electrolyte, but are mainly by FSI^- anions in DX-based electrolyte. It is also worth noting that the schematic diagram in Figure 4c of main manuscript is to illustrate the solvation structure within the EDL to explain the AFM force curve results, the real solvation structure within a confined liquid space[43-45] under the high internal electric field of EDL might deviate from the bulk state[24, 46]. This is still an open question that requires more in-depth experiment and MD simulation studies.

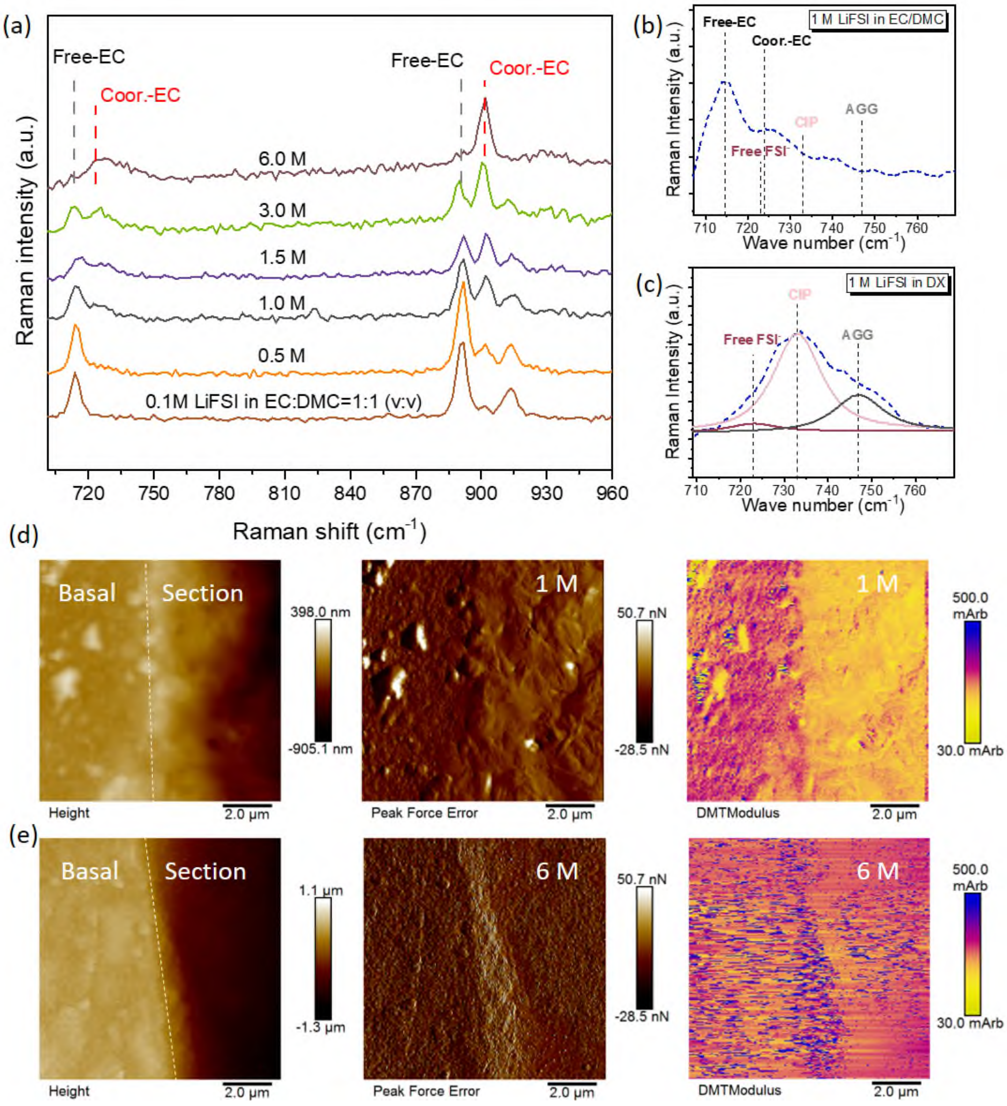

Supplementary Fig. 18 Effects of salt concentration on solvation structure and SEI nanomechanical property. (a) Raman spectra of LiFSI in EC: DMC=1:1 electrolyte with different salt concentrations. Raman spectra showing the FSI anions coordination statutes in (b) 1M LiFSI in EC: DMC=1:1 and (c) 1M LiFSI in DX electrolyte. Surface morphology, peak-force error, and DMT nano-mechanical modulus of SEI layers formed in (d) 1M and (e) 6M LiFSI in EC: DMC=1:1electrolyte. Unit (a. u.) denotes arbitrary scale.

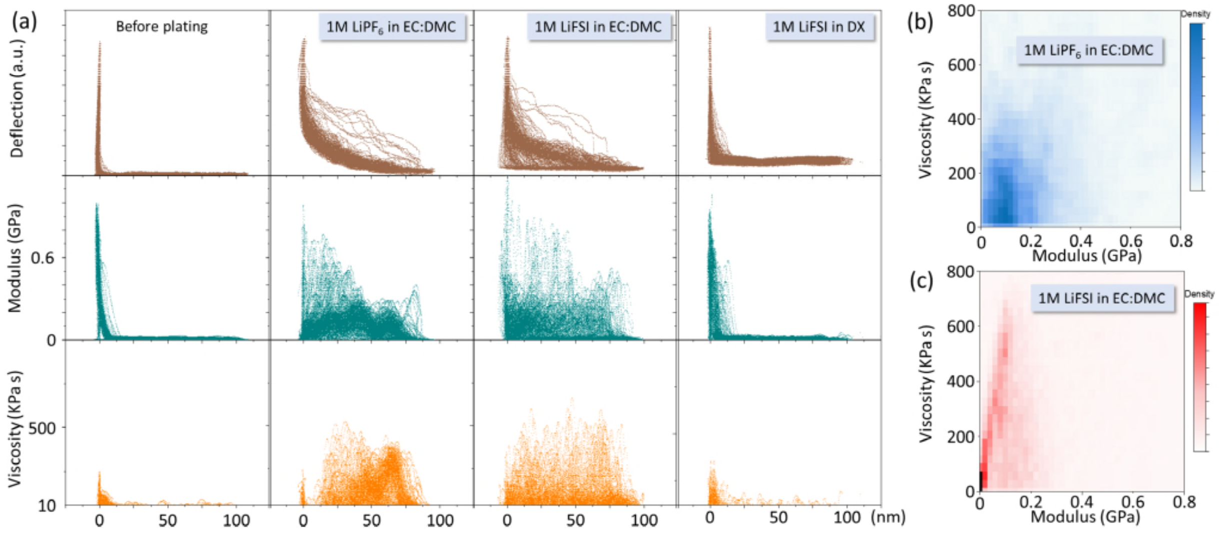

Supplementary Fig. 19 Nanomechanical properties of SEI formed in three different electrolytes (1M LiPF6 in EC: DMC, 1M LiFSI in EC: DMC and 1M LiFSI in DX) on lithium metal surface. (a) Tip deflection and 3D-NRM measured effective shear modulus, $G(Z)$, and viscosity, $\mu(Z)$, spectra on lithium metal before and after the 1st plating in different electrolytes. Each spectrum is an overlapping record of 256 tip-approach curves, the sample surface corresponded to zero on the horizontal axis. (b, c) The 2D G- μ plot of the SEI mechanical properties after 1st plating in (b) $LiPF_6$ and (c) LiFSI electrolytes. The plating current density is about 0.5 mA/cm^2. Unit (a. u.) denotes arbitrary scale.

Supplementary Note 12. To further understand how the cation solvation structure affects the SEI structure in the anode electrode with a homogenous surface structure, we quantified the SEI nano-mechanical properties on surface of the platting/stripping based metallic anode in the different electrolytes.

Fig. 19a shows the deflection and 3D-NRM effective modulus and viscosity spectra on the lithium metal surface before (open circuit potential) and after the first plating in the different electrolytes. Before the plating, the deflection spectrum consists mainly of a horizontal line (no mechanical force acting on the tip away from the surface) and a vertical line (solid-solid tip-anode contact), indicating a relatively ‘clean’ electrode surface. However, after the first plating and SEI formation, the deflection spectra in the two EC-based electrolytes (1 M $LiPF_6$ or LiFSI in EC: DMC) both show a noticeable mechanical response away from the metallic anode surface. These derive from the compressible surface SEI layer which was pressed by the tip during the ramping approach towards the electrode surface with some possible contribution from the newly deposited lithium nano-crystalline. The average modulus and viscosity spectra correspond to the average G and μ around 0.15 GPa and 200 kPa·s, respectively. The 2D correlation plot of these two surface mechanical properties G and μ are shown in Figs. 19b and 19c. Importantly, the deflection spectra on the lithium surface after plating in DX-based electrode does not show a compressible surface layer, and the derivative of the nanomechanical force vs distance dF/dZ is significantly larger compared to the ones formed on the EC-based electrolyte. This indicates a stiffer and compact surface structure formed on the lithium metal anode surface after the 1st plating in the 1M LiFSI in DX solvent. Mechanical spectra measured on the lithium metal surface support that the electrolyte decomposition layer on the plating-based lithium metal anode is highly dependent on the solvent rather than the salt anion (PF_6 or FSI). The decomposition product formed in the DX-based electrolyte has higher mechanical stiffness which indicates a more inorganic decomposition product from the FSI^- anions. The relatively soft surface layer formed on the EC based electrolyte could attribute to the preferential reduction of the EC/DMC molecular within the Li^+ solvents sheath. The above considerations suggest (and are supported by the explanations below based on the controlled experiments) that the competitive coordination effect of solvent and anion toward cations, which determines the lithium-ion solvation structures, is more important than the sole effect of different anion (PF_6 and FSI) chemistries in terms of electrolyte decomposition paths (solvent or anion dominated).

This can be also explained by the Raman spectra of LiFSI in EC/DMC as shown in Figure 18a. The strong EC-Li coordination peaks, at around 723 cm^{-1} and 915 cm^{-1}, are observed in LiFSI in EC/DMC electrolytes at concentrations ranging from 0.1 to 6 M, indicating the strong coordination ability of high dielectric constant EC solvent compared with FSI anions[27]. This solvent preferential coordination is similar to the 1M $LiPF_6$ in EC/DMC as proved in the Raman spectrum in Figure 5 of the main manuscript. Additionally, comparing Figure 18b with 18c, the S-F-S stretch peak derived from the aggregated Li-FSI coordination at around 747 cm^{-1} does not exist in EC/DMC based electrolyte, confirming that the lithium ions in 1M LiFSI mixed with EC/DMC electrolyte mainly form uncoordinated free ion and solvent share/separated ion-pairs, rather than the ion aggregates as in weakly solvating DX-based electrolyte. Namely, Li-ions in 1M LiFSI in EC/DMC are mainly solvated by solvent molecules in the first solvation shell, similar to 1M $LiPF_6$ in EC/DMC electrolyte.

As a result, the SEI measurement by AFM (Figure 18d) found that, similar to the SEI structures formed in 1M $LiPF_6$ in EC/DMC, a soft organic-rich SEI layer in the sample section and stiff inorganic-rich SEI layer on the basal plane are also formed in the sample cycled in 1M LiFSI in EC/DMC. This suggests that it is the solvation structure (competition effects of solvents and anions), rather than the anion species, that dominates the preferential electrolyte decomposition process in these electrolyte systems[47, 48].

More significantly, another proof of the importance of solvation structure is as follows: In a relatively high concentration (6M) LiFSI in EC/DMC electrolyte, the SEI layer becomes a uniform stiff layer on both sample section and basal plane as shown in Figure 18e. This is because when the concentration reaches 6M (LiFSI: EC: DMC≈1:1.3:0.9), almost every oxygen atom in EC is coordinating with Li-ions according to the Raman spectrum in Figure 18a, but this is not enough to “surround” all Li-ions and screen the electrostatic charges. In this case, FSI anions inevitably participate in the first solvation shell. This increases the possibility of anion reduction on the electrode surface and results in more anion-derived SEI interfacial chemistries, similar to the weakly solvating electrolyte using DX solvent.

Overall, this consideration and experiments allow to presume that the competition between the anion and solvents in the first solvation shell can be tailored by changing the strongly/weakly solvating solvent species and salt concentration, which plays a more important role in determining the SEI formations. By modulating the dominating coordinators with lithium-ions, one can control the SEI interfacial chemistries by guiding the electrolyte decomposition paths.

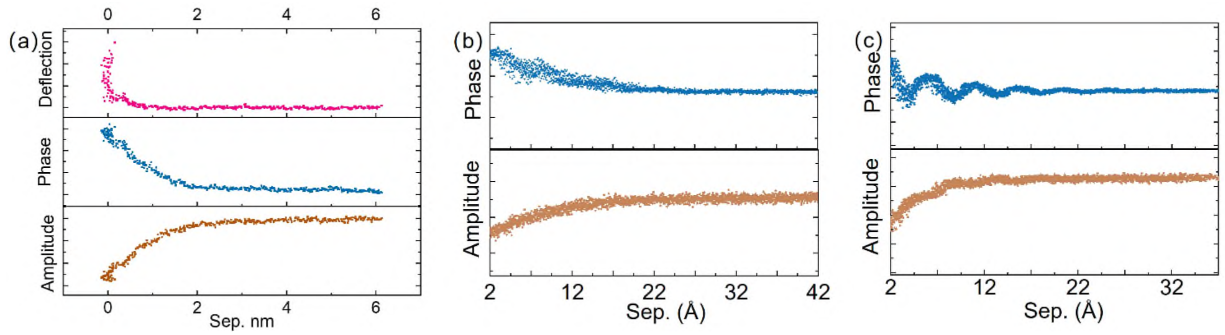

Supplementary Fig. 20 Tip-surface distance calibration in amplitude modulation mode Force spectroscopy. (a) Typical tip-sample separation calibration curves using the deflection signal. The overlapped tip-tapping amplitude and phase approaching curves in (b) EC based electrolyte and (c) DX-based electrolyte. Tip: ARROW-UHFAuD-10, the first resonance frequency in liquid f_0~656.5 kHz, force constant k~10-30 N/m, drive amplitude 50-100 pm, tip ramp size 5 nm.

Supplementary Note 13. In the force volume ramp mode, a triangular excitation AC voltage was applied to the z piezo to generate the ramp movement of the tip. The ramp cycles are conducted in a scan size of 5 nm in the X-Y plane in Force-volume mode. During the ramp cycles, the AFM force spectra were operated in amplitude modulation mode by using the amplitude as the feedback control. The force curves were performed on the HOPG basal plane at around 2.7-2.5 V vs lithium reference electrode at which no SEI formation is observed according to CV measurements. The force-separation (FS) curves are reconstructed based on the force reconstruction methods developed for amplitude modulation AFM according to the Equation [49, 50]

$$F(S) = -\frac{\partial}{\partial S}\int_{S}^{S+2A} \frac{kA^{\frac{3}{2}}}{\sqrt{2(Z-S)}}\left[\frac{A_d\cos(\phi)}{A} - \frac{f_0^2-f_d^2}{f_0^2}\right]dZ \qquad (9)$$

Where $F(S)$ is the tip-EDL interaction force at the separation position S, A and ϕ are the tip amplitude and phase shift recorded in the ramp experiments, Z is the position of the tip apex, f_0 is the tip free resonance in liquid, f_d is the tip drive frequency, A_d is the tip drive amplitude, and k is the force constant of the cantilever. In amplitude modulation AFM, we record the average cantilever deflection, the oscillation amplitude A and the phase shift ϕ during each ramp cycle simultaneously. FS curves were computed from the dynamic observables A and ϕ (Fig. 20a) as a function of the calibrated z-position (separation). The tip-sample separation was calibrated by the deflection as shown in Fig. 20a. To increase the signal-to-noise ratio in the FS curves, the value of the tip force was obtained by overlapping and averaging the values of the observables for the different X-Y positions at the same z position as shown in Figs. 20b and 20c.

Supplementary Note 14. The detailed numbers of each species were calculated according to the densities, concentration and volume ratio of solvents in the electrolyte. As shown in Figs. 21a, b, the volume of simulation box is around 2.60×10^{-19} cm^3. For the 1M concentration electrolytes, the simulation box contains about 2.60×10^{-22} mole salt molecules, which corresponds to about 156 $LiPF_6$ or LiFSI. Since the electrolyte is dilute, we ignored the volume changes of the simulation box before and after adding salt. (This results on the error of less than 1% for these values, see the results in Supplementary Table 1 below) The volume ratio of EC: DMC is 1:1, therefore taking the density of 1.33±0.01 g/cm^3 for EC and 1.07±0.01 g/cm^3 for DMC at room temperature, the mass in the simulation box will be around for EC 1.72-1.74 × 10^{-19} g and for DMC 1.38-1.40 × 10^{-19} g, corresponding to the numbers of around 1175-1189 and 920-936 for EC and DMC molecules, respectively. The molecule numbers within this range are in an acceptable error range. Similarly, the number of DX in the simulation box and is around 1638, by using a room temperature density value of 1.03 g/cm^3.

For a more precise estimation using the final density of the commercial electrolyte (1.3634 g/mL for $LiPF_6$ in EC: DMC=1:1 (v:v) electrolyte), the number of $LiPF_6$ salt, EC, and DMC are determined as around 156.52, 1195.63 and 935.63, respectively (see the detailed calculation parameter in Supplementary Table 1). The numbers we used for $LiPF_6$, EC, and DMC are 156, 1187, and 934 respectively, which results in the error smaller than 1% using this more precise estimation.

These densities obtained from the experiment values were checked by NPT method to verify the equilibrium states of our simulation system, the results are shown in Figs. 21c-f. The errors between the simulation electrolyte density and experimental values are less than 8 % for both EC- and DX-based electrolyte, indicating that our simulation system can reliably represent the experiment solvation structure characteristics.

Supplementary Table 1 Detailed parameters for determining the molecule numbers in the simulation box by the overall density of $LiPF_6$ in EC: DMC=1:1 (v:v) electrolyte

Species	Mass in the simulation box (g)	Molecules weight (g/mol)	Numbers in the simulation box
$LiPF_6$	3.95×10^{-20}	151.905	156.52
EC	1.749×10^{-19}	88.062	1195.63
DMC	1.400×10^{-19}	90.078	935.63
Total mass (g)	3.544×10^{-19}		

Note:

Salt concentration: 1 M mol/mL

Volume ratio of EC: DMC=1:1, therefore the mass ratio $\frac{m_{EC}}{m_{DMC}} = \frac{\rho_{DMC}}{\rho_{EC}} = \frac{1.07}{1.33} \approx 0.80$

Electrolyte density: 1.3634 g/mL (see: *https://www.sigmaaldrich.com/GB/en/product/aldrich/809357*)

Volume of box: 2.60×10^{-19} cm^3

Total mass: 3.544×10^{-19} g

Avogadro constant: 6.02×10^{-23}

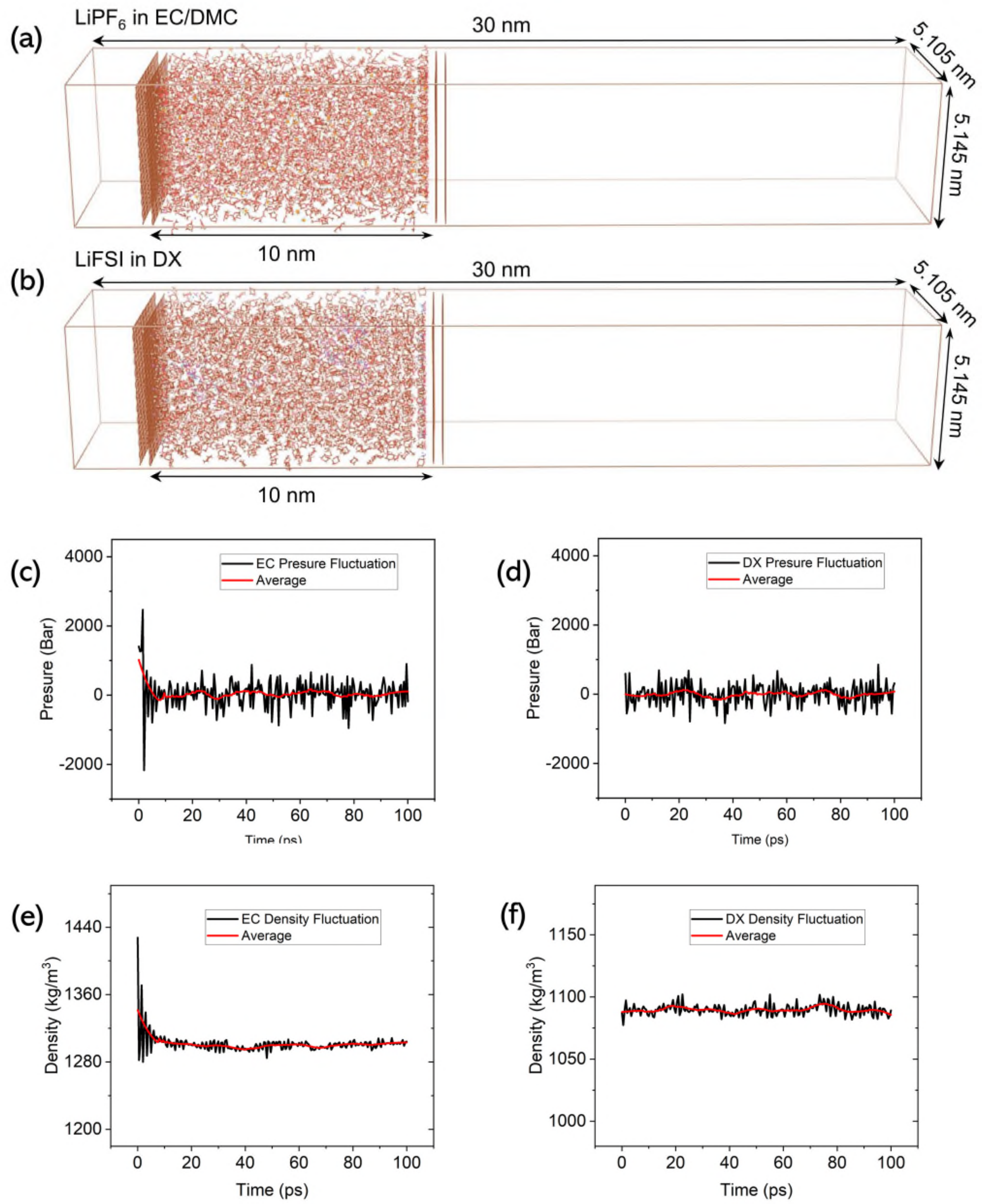

Supplementary Fig. 21 MD simulation box structure and equilibrium state checking. Visual representation of simulation box for (a) 156 $LiPF_6$, 1187 EC, and 934 DMC and 934 and (b) 156 LiFSI/1638 DX confined by two carbon (001). (c, d) Pressure and (e, f) density fluctuation curve during the equilibrium simulation of EC- and DX-based electrolytes.

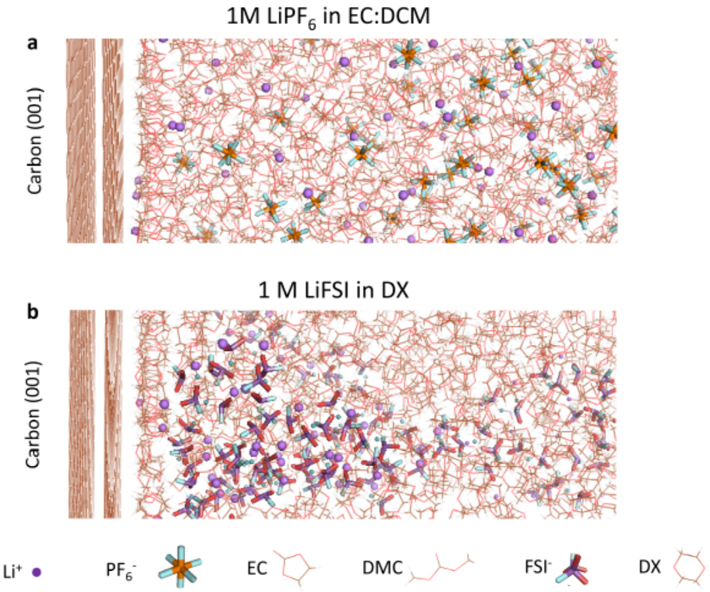

Supplementary Fig. 22 Snapshots of molecules/ions arrangement near the negatively charged electrode surface in two electrolytes by MD simulations. (a) 1M LiPF6 in EC: DMC, (b) 1M LiFSI in DX.

References:

1. Pan, H.; Chen, Y.; Pang, W.; Sun, H.; Li, J.; Lin, Y.; Kolosov, O.; Huang, Z., Complementary sample preparation strategies (PVD/BEXP) combining with multifunctional SPM for the characterizations of battery interfacial properties. *MethodsX* **2021,** *8*, 101250.
2. Robson, A. J.; Grishin, I.; Young, R. J.; Sanchez, A. M.; Kolosov, O. V.; Hayne, M., High-Accuracy Analysis of Nanoscale Semiconductor Layers Using Beam-Exit Ar-Ion Polishing and Scanning Probe Microscopy. *Acs Applied Materials & Interfaces* **2013,** *5* (8), 3241-3245.
3. Kolosov, O.; Briggs, G. A. D.; Yamanaka, K.; Arnold, W., Nanoscale imaging of mechanical properties by ultrasonic force microscopy (UFM). In *Acoustical Imaging, Vol 22*, Tortoli, P. M. L., Ed. 1996; Vol. 22, pp 665-668.
4. Kolosov, O., UFM shakes out the details at the nanoscopic scale. *Mater World* **1998,** *6* (12), 753-754.
5. Sole, C.; Drewett, N. E.; Hardwick, L. J., Insitu Raman study of lithium-ion intercalation into microcrystalline graphite. *Faraday Discuss.* **2014,** *172*, 223-237.
6. Liu, T.; Lin, L.; Bi, X.; Tian, L.; Yang, K.; Liu, J.; Li, M.; Chen, Z.; Lu, J.; Amine, K.; Xu, K.; Pan, F., In situ quantification of interphasial chemistry in Li-ion battery. *Nature nanotechnology* **2019,** *14* (1), 50-56.
7. Chen, Y.; Kolosov, O. V., Exploring solid-electrolyte-interphase in rechargeable batteries.
8. Chen, Y.; Pan, H.; Lin, C.; Li, J.; Cai, R.; Haigh, S. J.; Zhao, G.; Zhang, J.; Lin, Y.; Kolosov, O. V.; Huang, Z., Controlling Interfacial Reduction Kinetics and Suppressing Electrochemical Oscillations in Li4Ti5O12 Thin-Film Anodes. *Advanced Functional Materials* **2021,** *n/a* (n/a), 2105354.
9. O'Shea, S. J.; Welland, M. E.; Pethica, J. B., Atomic force microscopy of local compliance at solid—liquid interfaces. *Chemical Physics Letters* **1994,** *223* (4), 336-340.
10. Garcia, R., Nanomechanical mapping of soft materials with the atomic force microscope: methods, theory and applications. *Chemical Society Reviews* **2020,** *49* (16), 5850-5884.
11. Gao, Y.; Du, X.; Hou, Z.; Shen, X.; Mai, Y.-W.; Tarascon, J.-M.; Zhang, B., Unraveling the mechanical origin of stable solid electrolyte interphase. *Joule* **2021,** *5* (7), 1860-1872.
12. Palacio, M. L.; Bhushan, B., Normal and lateral force calibration techniques for AFM cantilevers. *Critical Reviews in Solid State and Materials Sciences* **2010,** *35* (2), 73-104.
13. Johnston, I. D.; McCluskey, D. K.; Tan, C. K. L.; Tracey, M. C., Mechanical characterization of bulk Sylgard 184 for microfluidics and microengineering. *J Micromech Microeng* **2014,** *24* (3), 035017.
14. Rolland, J. P.; Van Dam, R. M.; Schorzman, D. A.; Quake, S. R.; DeSimone, J. M., Solvent-

Resistant Photocurable “Liquid Teflon” for Microfluidic Device Fabrication. *Journal of the American Chemical Society* **2004,** *126* (8), 2322-2323.

15. <PIIS0006349594810112 (1).pdf>.

16. Jeong, S.-K.; Inaba, M.; Abe, T.; Ogumi, Z., Surface Film Formation on Graphite Negative Electrode in Lithium-Ion Batteries: AFM Study in an Ethylene Carbonate-Based Solution. *Journal of The Electrochemical Society* **2001,** *148* (9).

17. Jeong, S.-K.; Inaba, M.; Mogi, R.; Iriyama, Y.; Abe, T.; Ogumi, Z., Surface Film Formation on a Graphite Negative Electrode in Lithium-Ion Batteries: Atomic Force Microscopy Study on the Effects of Film-Forming Additives in Propylene Carbonate Solutions. *Langmuir* **2001,** *17* (26), 8281-8286.

18. Ding, J. F.; Xu, R.; Yao, N.; Chen, X.; Xiao, Y.; Yao, Y. X.; Yan, C.; Xie, J.; Huang, J. Q., Non-Solvating and Low-Dielectricity Cosolvent for Anion-Derived Solid Electrolyte Interphases in Lithium Metal Batteries. *Angewandte Chemie International Edition* **2021**.

19. Jiang, L. L.; Yan, C.; Yao, Y. X.; Cai, W.; Huang, J. Q.; Zhang, Q., Inhibiting Solvent Co-Intercalation in a Graphite Anode by a Localized High-Concentration Electrolyte in Fast-Charging Batteries. *Angewandte Chemie International Edition* **2020,** *60* (7), 3402-3406.

20. Yao, Y. X.; Chen, X.; Yan, C.; Zhang, X. Q.; Cai, W. L.; Huang, J. Q.; Zhang, Q., Regulating Interfacial Chemistry in Lithium-Ion Batteries by a Weakly Solvating Electrolyte**. *Angewandte Chemie International Edition* **2020,** *60* (8), 4090-4097.

21. Ming, J.; Cao, Z.; Wahyudi, W.; Li, M.; Kumar, P.; Wu, Y.; Hwang, J.-Y.; Hedhili, M. N.; Cavallo, L.; Sun, Y.-K.; Li, L.-J., New Insights on Graphite Anode Stability in Rechargeable Batteries: Li Ion Coordination Structures Prevail over Solid Electrolyte Interphases. *ACS Energy Letters* **2018,** *3* (2), 335-340.

22. Vatamanu, J.; Borodin, O.; Smith, G. D., Molecular Dynamics Simulation Studies of the Structure of a Mixed Carbonate/LiPF6 Electrolyte near Graphite Surface as a Function of Electrode Potential. *The Journal of Physical Chemistry C* **2011,** *116* (1), 1114-1121.

23. Xing, L.; Vatamanu, J.; Borodin, O.; Smith, G. D.; Bedrov, D., Electrode/Electrolyte Interface in Sulfolane-Based Electrolytes for Li Ion Batteries: A Molecular Dynamics Simulation Study. *The Journal of Physical Chemistry C* **2012,** *116* (45), 23871-23881.

24. Rakov, D. A.; Chen, F.; Ferdousi, S. A.; Li, H.; Pathirana, T.; Simonov, A. N.; Howlett, P. C.; Atkin, R.; Forsyth, M., Engineering high-energy-density sodium battery anodes for improved cycling with superconcentrated ionic-liquid electrolytes. *Nature materials* **2020**.

25. Morita, M.; Asai, Y.; Yoshimoto, N.; Ishikawa, M., A Raman spectroscopic study of organic electrolyte solutions based on binary solvent systems of ethylene carbonate with low viscosity solvents which dissolve different lithium salts. *Journal of the Chemical Society, Faraday Transactions* **1998,** *94* (23), 3451-3456.

26. Mukai, K.; Inoue, T.; Kato, Y.; Shirai, S., Superior Low-Temperature Power and Cycle Performances of Na-Ion Battery over Li-Ion Battery. *ACS Omega* **2017,** *2* (3), 864-872.

27. Uchida, S.; Ishikawa, M., Lithium bis(fluorosulfonyl)imide based low ethylene carbonate content electrolyte with unusual solvation state. *Journal of Power Sources* **2017,** *359*, 480-486.

28. Haneke, L.; Frerichs, J. E.; Heckmann, A.; Lerner, M. M.; Akbay, T.; Ishihara, T.; Hansen, M. R.; Winter, M.; Placke, T., Editors’ Choice—Mechanistic Elucidation of Anion Intercalation into Graphite from Binary-Mixed Highly Concentrated Electrolytes via Complementary 19F MAS NMR and XRD Studies. *Journal of The Electrochemical Society* **2020,** *167* (14).

29. Xu, K., Electrolytes and interphases in Li-ion batteries and beyond. *Chem Rev* **2014,** *114* (23), 11503-618.

30. Yamada, Y.; Sagane, F.; Iriyama, Y.; Abe, T.; Ogumi, Z., Kinetics of Lithium-Ion Transfer at the Interface between Li0.35La0.55TiO3 and Binary Electrolytes. *The Journal of Physical Chemistry C* **2009,** *113* (32), 14528-14532.

31. Seo, D. M.; Reininger, S.; Kutcher, M.; Redmond, K.; Euler, W. B.; Lucht, B. L., Role of Mixed Solvation and Ion Pairing in the Solution Structure of Lithium Ion Battery Electrolytes. *The Journal of Physical Chemistry C* **2015,** *119* (25), 14038-14046.

32. Ponnuchamy, V.; Mossa, S.; Skarmoutsos, I., Solvent and Salt Effect on Lithium Ion Solvation and Contact Ion Pair Formation in Organic Carbonates: A Quantum Chemical Perspective. *The Journal of Physical Chemistry C* **2018,** *122* (45), 25930-25939.

33. Zhang, Y.; Su, M.; Yu, X.; Zhou, Y.; Wang, J.; Cao, R.; Xu, W.; Wang, C.; Baer, D. R.; Borodin, O.; Xu, K.; Wang, Y.; Wang, X. L.; Xu, Z.; Wang, F.; Zhu, Z., Investigation of Ion-Solvent

Interactions in Nonaqueous Electrolytes Using in Situ Liquid SIMS. *Analytical chemistry* **2018,** *90* (5), 3341-3348.

34. Bogle, X.; Vazquez, R.; Greenbaum, S.; Cresce, A.; Xu, K., Understanding Li(+)-Solvent Interaction in Nonaqueous Carbonate Electrolytes with (17)O NMR. *J Phys Chem Lett* **2013,** *4* (10), 1664-8.

35. Li, Q.; Cao, Z.; Wahyudi, W.; Liu, G.; Park, G.-T.; Cavallo, L.; Anthopoulos, T. D.; Wang, L.; Sun, Y.-K.; Alshareef, H. N.; Ming, J., Unraveling the New Role of an Ethylene Carbonate Solvation Shell in Rechargeable Metal Ion Batteries. *ACS Energy Letters* **2020,** *6* (1), 69-78.

36. Chen, X.; Zhang, Q., Atomic Insights into the Fundamental Interactions in Lithium Battery Electrolytes. *Accounts Chem. Res.* **2020,** *53* (9), 1992-2002.

37. Chen, X.; Yao, N.; Zeng, B.-S.; Zhang, Q., Ion–solvent chemistry in lithium battery electrolytes: From mono-solvent to multi-solvent complexes. *Fundamental Research* **2021,** *1* (4), 393-398.

38. Yao, Y. X.; Chen, X.; Yan, C.; Zhang, X. Q.; Cai, W. L.; Huang, J. Q.; Zhang, Q., Regulating Interfacial Chemistry in Lithium-Ion Batteries by a Weakly Solvating Electrolyte*. *Angewandte Chemie* **2021,** *60* (8), 4090-4097.

39. Borowski, P.; Gac, W.; Pulay, P.; Woliński, K., The vibrational spectrum of 1,4-dioxane in aqueous solution – theory and experiment. *New Journal of Chemistry* **2016,** *40* (9), 7663-7670.

40. Shi, P.; Zheng, H.; Liang, X.; Sun, Y.; Cheng, S.; Chen, C.; Xiang, H., A highly concentrated phosphate-based electrolyte for high-safety rechargeable lithium batteries. *Chem. Commun.* **2018,** *54* (35), 4453-4456.

41. Kimura, K.; Motomatsu, J.; Tominaga, Y., Correlation between Solvation Structure and Ion-Conductive Behavior of Concentrated Poly(ethylene carbonate)-Based Electrolytes. *The Journal of Physical Chemistry C* **2016,** *120* (23), 12385-12391.

42. Li, L.; Zhou, S.; Han, H.; Li, H.; Nie, J.; Armand, M.; Zhou, Z.; Huang, X., Transport and Electrochemical Properties and Spectral Features of Non-Aqueous Electrolytes Containing LiFSI in Linear Carbonate Solvents. *Journal of The Electrochemical Society* **2011,** *158* (2).

43. Han, M.; Zhang, R.; Gewirth, A. A.; Espinosa-Marzal, R. M., Nanoheterogeneity of LiTFSI Solutions Transitions Close to a Surface and with Concentration. *Nano letters* **2021,** *21* (5), 2304-2309.

44. Benaglia, S.; Uhlig, M. R.; Hernandez-Munoz, J.; Chacon, E.; Tarazona, P.; Garcia, R., Tip Charge Dependence of Three-Dimensional AFM Mapping of Concentrated Ionic Solutions. *Physical review letters* **2021,** *127* (19), 196101.

45. Uhlig, M. R.; Garcia, R., In Situ Atomic-Scale Imaging of Interfacial Water under 3D Nanoscale Confinement. *Nano letters* **2021,** *21* (13), 5593-5598.

46. Mozhzhukhina, N.; Flores, E.; Lundstrom, R.; Nystrom, V.; Kitz, P. G.; Edstrom, K.; Berg, E. J., Direct Operando Observation of Double Layer Charging and Early Solid Electrolyte Interphase Formation in Li-Ion Battery Electrolytes. *J Phys Chem Lett* **2020,** *11* (10), 4119-4123.

47. Lei, S.; Zeng, Z.; Liu, M.; Zhang, H.; Cheng, S.; Xie, J., Balanced solvation/de-solvation of electrolyte facilitates Li-ion intercalation for fast charging and low-temperature Li-ion batteries. *Nano Energy* **2022,** *98*, 107265.

48. Zhang, L.; Chen, Y., Electrolyte solvation structure as a stabilization mechanism for electrodes. *Energy Materials* **2021,** *1* (1), 100004.

49. Hölscher, H., Quantitative measurement of tip-sample interactions in amplitude modulation atomic force microscopy. *Applied Physics Letters* **2006,** *89* (12).

50. Calò, A.; Domingo, N.; Santos, S.; Verdaguer, A., Revealing Water Films Structure from Force Reconstruction in Dynamic AFM. *The Journal of Physical Chemistry C* **2015,** *119* (15), 8258-8265.
