# Supplementary material for: Nanoarchitecture factors of solid electrolyte interphase formation via 3D nano-rheology microscopy and surface force-distance spectroscopy
Source: Nat Commun. 2023 Mar 10;14:1321. doi: 10.1038/s41467-023-37033-7 (PMC10006426; doi:10.1038/s41467-023-37033-7)
Supplement: Supplementary file 3 — Description of Additional Supplementary Files [file 41467_2023_37033_MOESM3_ESM.pdf]

Description of Additional Supplementary Items

Title: Supplementary Movie 1:

Description: 3D SECTION VIEW OF EDGE PLAN SEI_1

Title: Supplementary Movie 2:

Description: 3D SECTION VIEW OF BASAL PLAN SEI_2

Title: Supplementary Movie 3

Description: SELF-HEALING OF SEI LAYER AFTER NANOSCRATCHING

Title: Supplementary Movie 4:

Description: COMSOL SIMULATION OF TIP INDENTATION IN PDMS
