# Supplementary figures and images for: Nanoarchitecture factors of solid electrolyte interphase formation via 3D nano-rheology microscopy and surface force-distance spectroscopy
Source: Nat Commun. 2023 Mar 10;14:1321. doi: 10.1038/s41467-023-37033-7 (PMC10006426; doi:10.1038/s41467-023-37033-7)

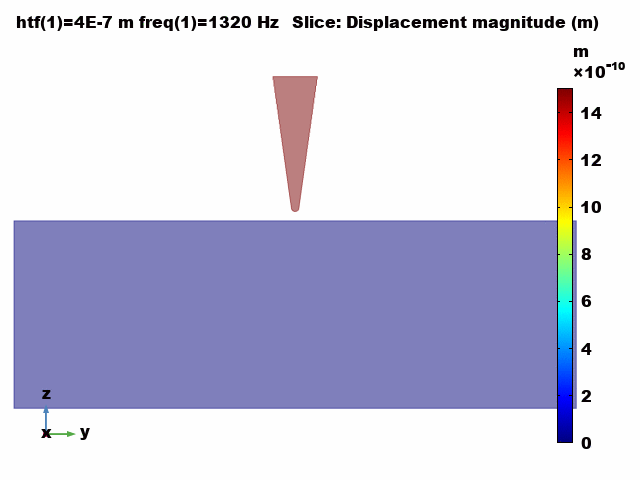

Supplement: Supplementary file 7 — Supplementary Movie 4. [file 41467_2023_37033_MOESM7_ESM.gif]
